# Store-Operated Ca^2+^ Entry Is Remodelled and Controls *In Vitro* Angiogenesis in Endothelial Progenitor Cells Isolated from Tumoral Patients

**DOI:** 10.1371/journal.pone.0042541

**Published:** 2012-09-25

**Authors:** Francesco Lodola, Umberto Laforenza, Elisa Bonetti, Dmitry Lim, Silvia Dragoni, Cinzia Bottino, Hwei Ling Ong, Germano Guerra, Carlo Ganini, Margherita Massa, Mariangela Manzoni, Indu S. Ambudkar, Armando A. Genazzani, Vittorio Rosti, Paolo Pedrazzoli, Franco Tanzi, Francesco Moccia, Camillo Porta

**Affiliations:** 1 Department of Biology and Biotechnology “Lazzaro Spallanzani”, University of Pavia, Pavia, Italy; 2 Section of Human Physiology, Department of Molecular Medicine, University of Pavia, Pavia, Italy; 3 Clinical Epidemiology Laboratory Fondazione IRCCS Policlinico San Matteo, Pavia, Italy; 4 Department of Pharmaceutical Sciences, University of Eastern Piedmont “Amedeo Avogadro”, Novara, Italy; 5 Secretory Physiology Section, Molecular Physiology and Therapeutics Branch, National Institute of Dental and Craniofacial Research, National Institutes of Health, Bethesda, Maryland, United States of America; 6 Department of Health Sciences, University of Molise, Campobasso, Italy; 7 Medical Oncology IRCCS Policlinico San Matteo, Pavia, Italy; 8 Laboratory of Biotechnology, Fondazione IRCCS Policlinico San Matteo, Pavia, Italy; University of Florida, United States of America

## Abstract

**Background:**

Endothelial progenitor cells (EPCs) may be recruited from bone marrow to sustain tumor vascularisation and promote the metastatic switch. Understanding the molecular mechanisms driving EPC proliferation and tubulogenesis could outline novel targets for alternative anti-angiogenic treatments. Store-operated Ca^2+^ entry (SOCE), which is activated by a depletion of the intracellular Ca^2+^ pool, regulates the growth of human EPCs, where is mediated by the interaction between the endoplasmic reticulum Ca^2+^-sensor, Stim1, and the plasmalemmal Ca^2+^ channel, Orai1. As oncogenesis may be associated to the capability of tumor cells to grow independently on Ca^2+^ influx, it is important to assess whether SOCE regulates EPC-dependent angiogenesis also in tumor patients.

**Methodology/Principal Findings:**

The present study employed Ca^2+^ imaging, recombinant sub-membranal and mitochondrial aequorin, real-time polymerase chain reaction, gene silencing techniques and western blot analysis to investigate the expression and the role of SOCE in EPCs isolated from peripheral blood of patients affected by renal cellular carcinoma (RCC; RCC-EPCs) as compared to control EPCs (N-EPCs). SOCE, activated by either pharmacological (i.e. cyclopiazonic acid) or physiological (i.e. ATP) stimulation, was significantly higher in RCC-EPCs and was selectively sensitive to BTP-2, and to the trivalent cations, La^3+^ and Gd^3+^. Furthermore, 2-APB enhanced thapsigargin-evoked SOCE at low concentrations, whereas higher doses caused SOCE inhibition. Conversely, the anti-angiogenic drug, carboxyamidotriazole (CAI), blocked both SOCE and the intracellular Ca^2+^ release. SOCE was associated to the over-expression of Orai1, Stim1, and transient receptor potential channel 1 (TRPC1) at both mRNA and protein level The intracellular Ca^2+^ buffer, BAPTA, BTP-2, and CAI inhibited RCC-EPC proliferation and tubulogenesis. The genetic suppression of Stim1, Orai1, and TRPC1 blocked CPA-evoked SOCE in RCC-EPCs.

**Conclusions:**

SOCE is remodelled in EPCs from RCC patients and stands out as a novel molecular target to interfere with RCC vascularisation due to its ability to control proliferation and tubulogenesis.

## Introduction

An increase in intracellular Ca^2+^ concentration ([Ca^2+^]_i_) occurs in all cell types following the activation of either G-protein coupled receptors or tyrosine kinase receptors [1], [2]. Agonist stimulation leads to the activation of various isoforms of the membrane-bound enzyme phospholipase C (PLC), which cleaves the lipid precursor phosphatidylinositol-4,5-bisphosphate (PIP_2_) to yield diacylglycerol (DAG) and inositol-1,4,5-trisphosphate (InsP_3_) [1]. InsP_3_ liberates Ca^2+^ stored within the endoplasmic reticulum (ER) by binding to its inositol-1,4,5-trisphosphate (InsP_3_)-sensitive receptors (InsP_3_Rs) [1]. The consequent drop in ER Ca^2+^ content signals the activation of a Ca^2+^-permeable route in the plasma membrane (PM), namely the store-operated Ca^2+^ entry (SOCE) pathway, which gates the subsequent Ca^2+^ inflow from extracellular space [1], [3]. SOCE is mediated by the physical coupling between the Ca^2+^-sensor, Stromal Interaction Molecule-1 (Stim1), on the ER membrane and the channel protein, Orai1, on the PM [3]. Stim1 is a single-pass transmembrane protein endowed with a Ca^2+^-binding EF domain on the amino-terminal ER luminal portion. When ER Ca^2+^ concentration falls below a threshold level due to InsP_3_Rs-dependent Ca^2+^ release, Stim1 proteins rapidly redistribute to peripheral ER sites in close proximity to PM, where they aggregate into multiple puncta [3]. Orai1 serves as the pore-forming subunit of store-operated channels: on store depletion, Orai1 molecules cluster into the same puncta containing Stim1 proteins, which bind to and activate Orai1 itself [3]. A number of studies have, however, shown that transient receptor potential channel 1 (TRPC1) may either serve as store-dependent channels upon binding to Stim1 or participate to SOCE by forming a ternary complex with Stim1 and Orai1. In the latter case, Orai1 is essential for TRPC1 to become store-sensitive [3]. SOCE controls several functions, including ER refilling, gene expression, apoptosis, proliferation, migration, and differentiation [4], [5]. Since these processes are all relevant to tumor development and metastatization, it is not surprising that the components of the Ca^2+^ toolkit may be aberrantly expressed in cancer cells [6]–[10]. For instance, Orai1 is over-expressed in a number of breast cancer cell (BCC) lines, which display a significantly higher SOCE compared to control cells [11]. Similarly, a number of TRP channels, including TRPC6, undergo a dramatic remodelling in tumoral cells [6], [9]. Nevertheless, a number of studies have shown that oncogenesis may dramatically reduce or abrogate the need for Ca^2+^ influx in neoplastic cells, which become able to undergo DNA synthesis and proliferation even in absence of extracellular Ca^2+^ inflow [9], [12], [13].

**Figure 1:**
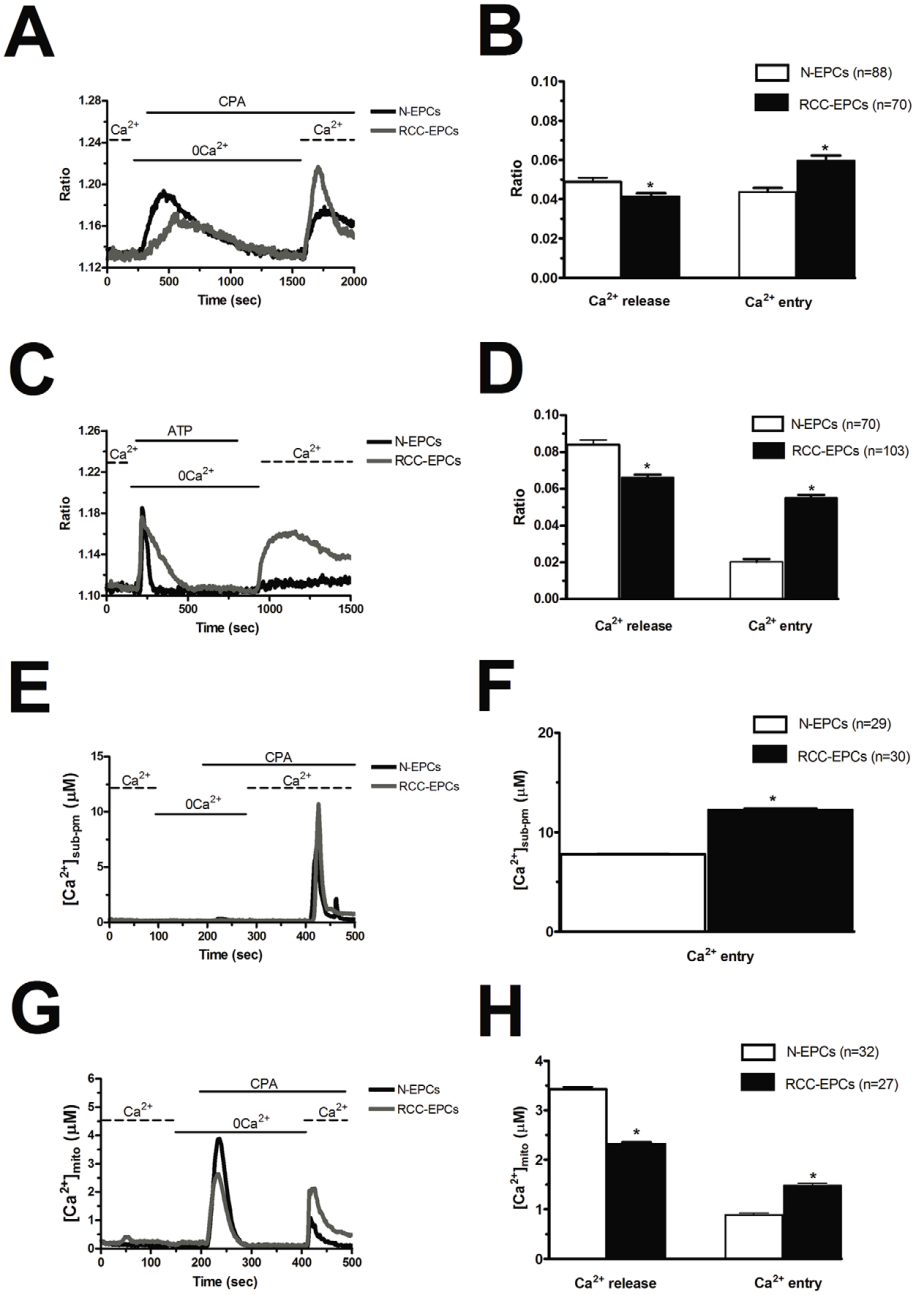
Store-dependent Ca^2+^ entry is higher in endothelial progenitor cells isolated from patients suffering from renal cellular carcinoma. A, during exposure to 0Ca^2+^ PSS, depletion of the intracellular Ca^2+^ stores resulted from addition of 10 µM CPA to the bathing medium. Subsequent replenishment of Ca^2+^ (1.5 mM) to the extracellular solution elicited a rise in [Ca^2+^]_i_ due to Ca^2+^ influx through open store-operated Ca^2+^ channels. Black and grey tracings depict the representative changes in [Ca^2+^]_i_ recorded from EPCs isolated from healthy volunteers (N-EPCs) and patients suffering from RCC (RCC-EPCs), respectively. The transient increase in [Ca^2+^]_i_ evoked by CPA under 0Ca^2+^ conditions decayed to the baseline with slower mono-exponential kinetics in RCC-EPCs as compared to N-EPCs (298.06±0.17 sec, n = 58, *vs*. 342.67±0.07 sec, n = 62, respectively). B, mean±SE of the amplitude of CPA-induced Ca^2+^ release and CPA-induced SOCE recorded from all EPCs isolated from both healthy donors (black bar) and RCC patients (white bar). The asterisk indicates p<0.05. C, cells perfused with ATP (100 µM) responded with a transient rise in cytosolic [Ca^2+^]_i_. After continued perfusion with 0Ca^2+^, restoration of external Ca^2+^ caused a sustained rise in cytosolic [Ca^2+^]_i_ due to SOCE activation. Black and grey tracings depict the changes in [Ca^2+^]_i_ recorded from representative EPCs isolated from healthy volunteers (N-EPCs) and patients suffering from RCC (RCC-EPCs), respectively. In these and the following figures, agonists and drugs were administered at the time indicated by the horizontal bars. The transient increase in [Ca^2+^]_i_ evoked by ATP under 0Ca^2+^ conditions decayed to the baseline with slower mono-exponential kinetics in RCC-EPCs as compared to N-EPCs (52.26±0.14 sec, n = 35, *vs*. 101.73±0.17 sec, n = 25, respectively). D, mean±SE of the amplitude of ATP-induced Ca^2+^ release and ATP-induced SOCE recorded from all EPCs isolated from both healthy donors (black bar) and RCC patients (white bar). The asterisk indicates p<0.05. Please note that the amplitude of SOCE was higher upon CPA, rather than ATP, stimulation in N-EPCs (see [27]). E, N-EPCs and RCC-EPCs were transduced by lentiviral particles expressing AEQ fused with SNAP25 (pm-AEQ). Intracellular stores were first depleted by challenging the cells with ATP (100 µM) in 0Ca^2+^, after which SOCE was triggered by restoring extracellular Ca^2+^ in the absence of the agonist. F, mean±SE of the magnitude of the luminescence emitted by pm-AEQ in both control cells (white bar) and RCC-EPCs (black bar). The asterisk indicates p<0.05. G, the cells were infected with lentiviral vector expressing AEQ targeted to the mitochondrial lumen (mit-AEQ) and the experiment conducted as depicted in Panels C and E. H, mean±SE of the amplitude of ATP-induced Ca^2+^ release and ATP-induced SOCE recorded in both N-EPC (black bar) and RCC-EPCs (white bar). The asterisk indicates p<0.05.

Renal cell carcinoma (RCC) accounts for 3.5% and 2.9% of malignant tumors in European men and women, respectively [14]. RCC tumorigenesis and metastatisation depend on the development of a highly intricate vascular network, which is due to an angiogenic process stimulated by the local release of growth factors and cytokines [15]. Accordingly, tyrosine kinase inhibitors are currently employed in RCC treatment, either alone or in combination with chemotherapy, although the majority of the patients develop drug resistance and exhibit toxic side-effects [16], [17]. These features dramatically hampered the efficacy of anti-angiogenic strategies and prompted the quest for alternative targets to adverse the vascular network supplying RCC with oxygen and nutrients [16]. In addition to the classic process of angiogenesis, tumor vascularisation may be supported by bone marrow (BM)-derived endothelial progenitor cells (EPCs) incorporating within sprouting neovessels [18]–[20]. This feature hinted at EPC inhibition as a novel therapeutic target to pursue along with anti-angiogenic treatments [21], [22]. Notably, EPC levels are higher in RCC patients [23] and in human RCC xenograft models [24]. Moreover, RCC developing within a kidney allograft may display Y-positive chromosome vessels within a Y-negative tumor [25]. We have recently shown that human EPCs isolated from peripheral blood (PB) of healthy donors express a SOCE pathway mediated by Stim1 and Orai1 [26], [27]. Blockade of SOCE affects EPC proliferation, migration and tubulogenesis [26]–[29] and might, therefore, provide novel means to impair tumoral neovascularisation if operative in EPCs isolated from tumoral patients [30]. A number of studies have, however, shown that the interaction between Stim1 and TRPC1 underlies SOCE in rat bone marrow-derived EPCs [30]. Here, we employed Ca^2+^ imaging, real-time reverse transcriptase polymerase chain reaction (qRT-PCR), gene silencing, western blot analysis, and functional assays to investigate the expression and the role of SOCE in EPCs isolated from RCC patients (RCC-EPCs). We demonstrated that RCC-EPCs display a greater SOCE, which correlated with the over-expression of Orai1, Stim1, and TRPC1 at both mRNA and protein level as compared to control cells (N-EPCs). We further showed that SOCE is selectively affected by the selective blockers, BTP-2, La^3+^, Gd^3+^, and 2-aminoethyl diphenylborinate (2-APB). Conversely, carboxyamidotriazole (CAI), an antiangiogenic drug which is in phase II clinical trials of metastatic RCC [31], [32], lacks selectivity in both cell types. Furthermore, we found that the pharmacological blockade of SOCE impairs RCC-EPC proliferation and tubulogenesis. Finally, we provided the evidence that the genetic suppression of Stim1, Orai1, and TRPC1 affects store-regulated Ca^2+^ influx in RCC-ECFCs. These proteins might, therefore, be conceived as novel molecular targets to adverse tumor neovascularization.

## Results

### SOCE is higher in EPCs isolated from RCC patients

The resting Ca^2+^ levels measured in RCC-EPCs and control cells (N-EPCs) were measured upon digital subtraction of the fluorescence background and were not significantly different, the average values of the Fura-2 ratio being 1.44±0.02, n = 137, and 1.41±0.01, n = 245, respectively. Store-operated Ca^2+^ entry in RCC-EPCs was evaluated by exploiting the classical “Ca^2+^ add-back” protocol [33]. SOCE activation is triggered by a signal generated by either pharmacological or physiological depletion of the intracellular Ca^2+^ pool. RCC-EPCs were first exposed to cyclopiazonic acid (CPA) (10 µM), a SERCA inhibitor, which prevents Ca^2+^ reuptake into ER and promotes Ca^2+^ efflux into the cytosol through ER leak channels. This manoeuvre results in depletion of ER Ca^2+^ reservoir and SOCE activation [27], [33]. In absence of extracellular Ca^2+^ (0Ca^2+^), CPA induced a transient elevation in intracellular Ca^2+^ concentration ([Ca^2+^]_i_) in RCC-EPCs due passive ER emptying (Fig. 1A). Replenishment of extracellular Ca^2+^ (1.5 mM) caused Ca^2+^ entry through opened store-operated channels and provoked a large increase in [Ca^2+^]_i_ (Fig. 1A). SOCE amplitude was significantly (p<0.05) higher in RCC-EPCs as compared to N-EPCs, whereas CPA-induced Ca^2+^ release, which reflects ER Ca^2+^ content, was significantly (p<0.05) lower in the former cells (Fig. 1A and Fig. 1B; see also Figs. S1A-S1C). RCC-EPCs were then exposed to ATP (100 µM), which stimulates P_2Y_ receptors to synthesize InsP_3_ and induces Ca^2+^ release from ER in N-EPCs [27]. On adding back extracellular Ca^2+^ to the bathing medium, ATP-stimulated cells displayed a robust increase in [Ca^2+^]_i_, which was indicative of SOCE. In agreement with CPA data, ATP-induced SOCE was significantly (p<0.05) higher in RCC-EPCs as compared to N-EPCs (Fig. 1C and Fig. 1D; see also Figs. S1D–S1F). The agonist was removed before Ca^2+^ restoration to the bath to prevent Ca^2+^ influx through second messengers-operated channels and P_2X_ receptors [27], [34]–[35]. That ATP-elicited Ca^2+^ inflow is mediated by SOCE is also indicated by the observation that no additional Ca^2+^ influx is detectable in both types of EPCs stimulated with ATP (100 µM) upon depletion of the InsP_3_-sensitive Ca^2+^ stores and full activation of SOCE with CPA (10 µM) (Fig. S2A and Fig. S2B). Therefore, the same Ca^2+^-permeable membrane pathway, namely SOCE, is engaged by both CPA and ATP. Notably, no Ca^2+^ influx was detected when the “Ca^2+^ add-back” protocol was carried out in the absence of either CPA or ATP (Fig. S2C and Fig. S2D). Collectively, these findings show that SOCE is expressed and up-regulated in RCC-EPCs. Similar to CPA, ATP-evoked Ca^2+^ transients in 0Ca^2+^ were significantly (p<0.05) smaller in RCC-EPCs than in N-EPCs (Fig. 1C). This feature was not only due to the lower ER Ca^2+^ content revealed by CPA experiments. qRT-PCR analysis demonstrated that the transcripts of all the three known InsP_3_R isoforms were down-regulated (or even absent, in the case of InsP_3_R-1) in RCC-EPCs (Fig. S3). Ongoing experiments in our lab are exploring this issue, whose assessment was beyond the goal of the present study.

As an alternative approach to estimate store-dependent Ca^2+^ influx in RCC-EPCs, we used aequorin (AEQ) Ca^2+^ measurements combined with lentiviral gene transfer technology. The N-EPCs and RCC-EPCs were transduced by lentiviral particles expressing AEQ fused with SNAP25 (pm-AEQ) which allows to measuring Ca^2+^ precisely beneath the plasma membrane. Fig. 1E shows that re-addition of Ca^2+^ to the bath after depletion of internal Ca^2+^ stores by ATP under 0Ca^2+^ conditions resulted in a significantly higher Ca^2+^ increase in the sub-plasmalemmal space of RCC-EPCs as compared to N-EPCs. The statistical analysis of these results has been provided in Fig. 1F. To explore if the increased SOCE may have repercussion on Ca^2+^ homeostasis within intracellular organelles, such as mitochondria, the cells were infected with a lentiviral vector expressing AEQ targeted to the mitochondrial lumen by using a cleavable leader sequence of cytochrome peroxidase subunit 8 (COX8, mit-AEQ). In line with Fura-2 experiments, stimulation with 100 µM ATP resulted in significantly lower Ca^2+^ transient in the mitochondrial lumen of RCC-EPCs as compared with control N-EPCs (Fig. 1G and Fig. 1H). When extracellular Ca^2+^ was then re-added to the cells, the Ca^2+^ transient in the mitochondrial lumen was much higher in RCC-EPCs as compared to N-EPCs (Fig. 1G and Fig. 1H), thereby reflecting the increase in SOCE in the former cells observed independently in Fura-2 and pm-AEQ experiments.

### The higher amplitude of SOCE in RCC-EPCs does not depend on differences in resting membrane potential

**Figure 2:**
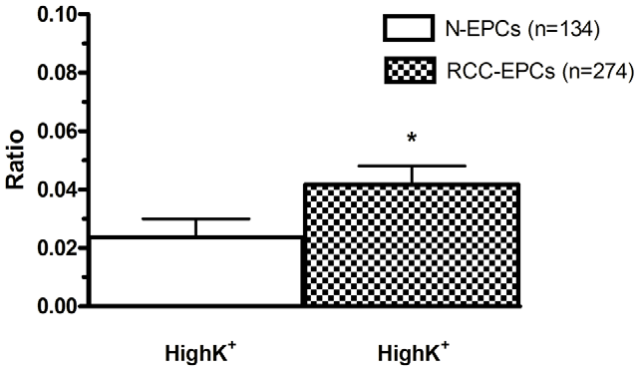
The amplitude of store-operated Ca^2+^ entry in endothelial progenitor cells isolated from patients affected by renal cellular carcinoma is still higher in the presence of a high-K^+^ extracellular solution. Mean±SE of CPA-evoked SOCE in N-EPCs and RCC-EPCs bathed in presence of an extracellular solution containing 100 mM KCl to clamp V_M_ at the same value in both cell types. CPA was applied at 10 µM. The asterisk indicates p<0.05 (p = 0.039).

The higher amplitude of store-dependent Ca^2+^ entry in RCC-EPCs could, in theory, be caused by a more hyperpolarized membrane potential (V_M_) as compared to control cells. A more negative V_M_ would enhance the electrochemical gradient driving Ca^2+^ inrush into the cells and lead to a more robust SOCE [36]. The endothelial V_M_ is mainly set by K^+^ conductances [36]. Therefore, to clamp N-EPCs and RCC-EPCs at the same V_M_ of about 0 mV, both types of cells were bathed in a solution containing 100 mM KCl (high-K^+^), as indicated by the Nernst equation for K^+^, E_K_  = (−RT/F)*ln([K^+^]_o_/[K^+^]_i_). Figure 2 shows that, under such conditions, CPA-elicited SOCE was still significantly higher in RCC-EPCs. Therefore, the larger height of SOCE in these cells is not attributable to a larger driving force for Ca^2+^ entry.

### Remodelling of Orai1, Stim1, and TRPC1 in RCC-EPCs

**Figure 3:**
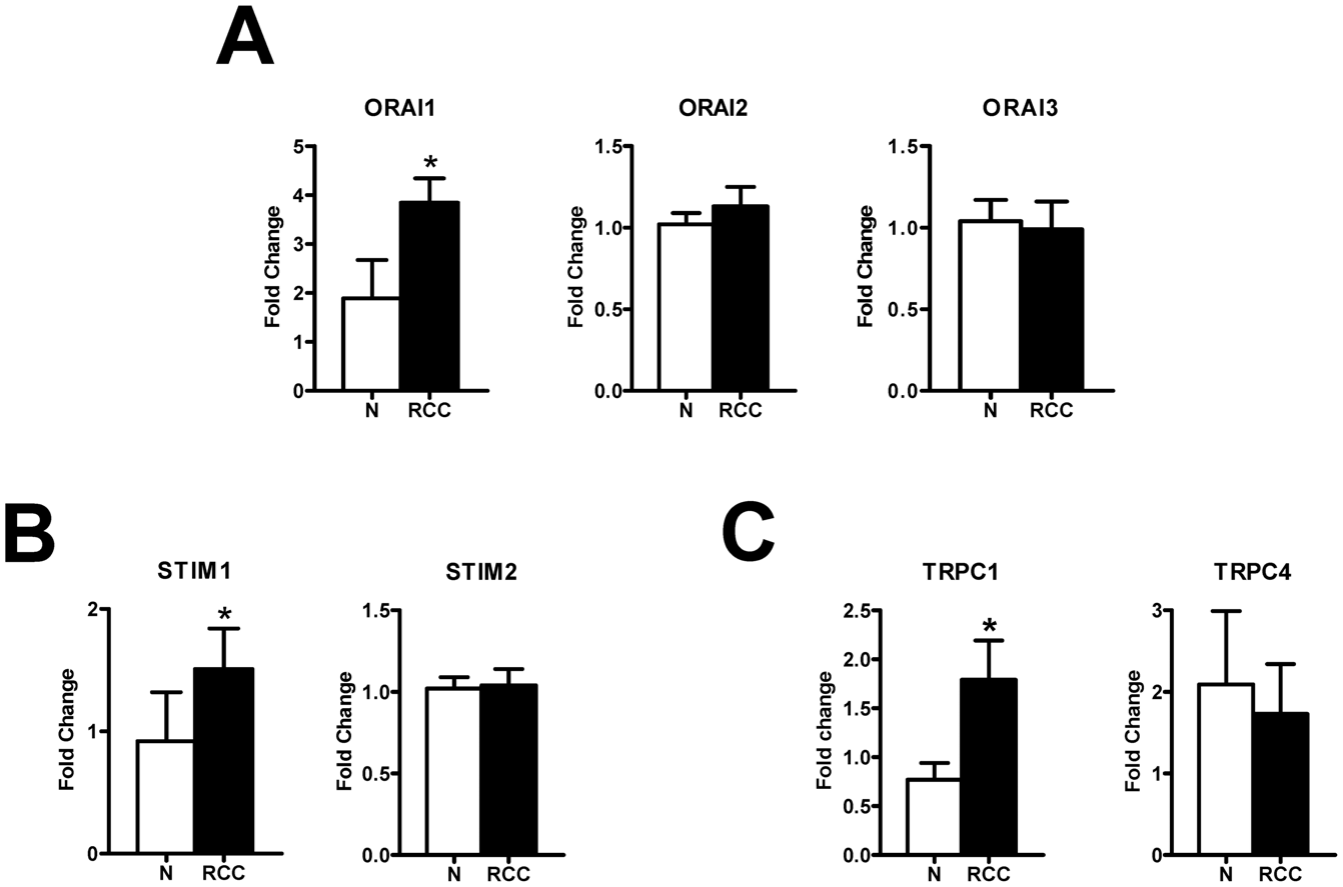
The expression of Orai1-3, Stim1-2, TRPC1 and TRPC4 in endothelial progenitor cells isolated from healthy donors and from patients suffering from renal cellular carcinoma. qRT-PCR showing increased expression of Orai1 (A), Stim1 (B), and TRPC1 (C) mRNA in RCC-EPCs compared to N-EPCs. Conversely, Orai2 (A), Orai3 (A), Stim2 (B), and TRPC4 (C) are not differently expressed in RCC-EPCs. Bars represent mean±SE of at least 4 different experiments each from different RNA extracts. The asterisk indicates p<0.05 *vs*. N-EPCs (ANOVA followed by Newman-Keuls' *Q* test). The PCR products were of the expected size: Orai1, 257 bp; Orai2, 334 bp; Orai3, 159 bp; Stim1, 347 bp; Stim2, 186 bp; TRPC1, 307 bp and TRPC4, 300 bp [27].

**Figure 4:**
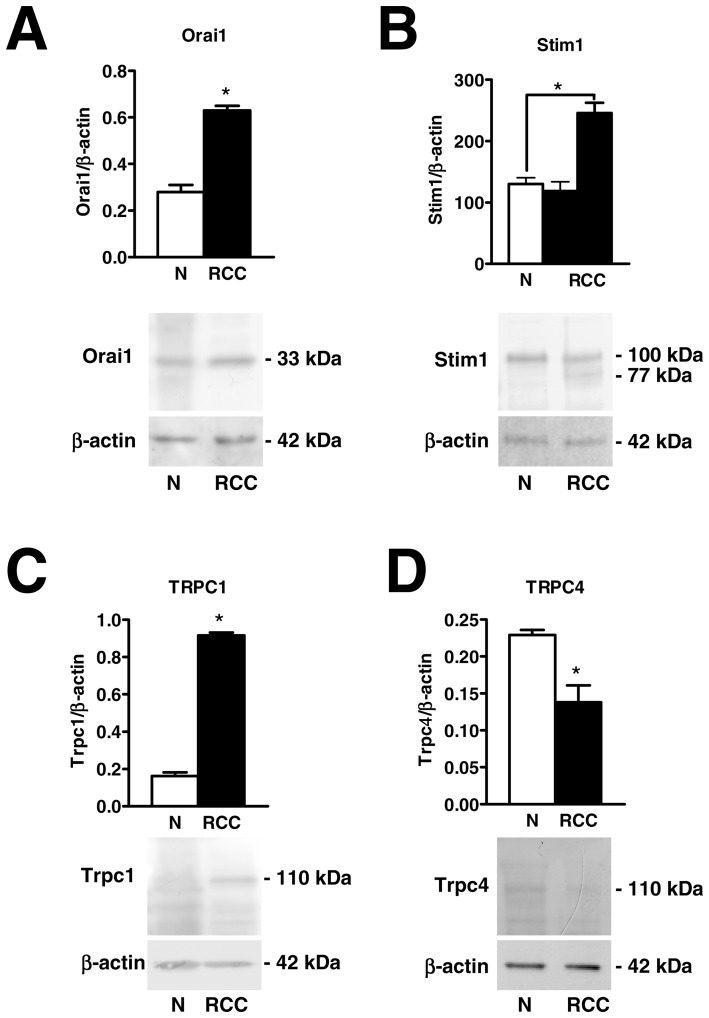
Orai1, Stim1, and TRPC1 proteins are over-expressed in endothelial progenitor cells isolated from patients suffering from renal cellular carcinoma. Western blot and densitometry depicting the significant elevation in Orai1 (A), Stim1 (B), and TRPC1 (C) proteins in RCC-EPCs as compared to N-EPCs. Conversely, TRPC4 protein (D) is down-regulated in RCC-EPCs. Blots for Orai1, Stim1, TRPC1 and TRPC4 representative of 4 different experiments are shown in the lower panel. Lanes were loaded with 20 μg of proteins. Major bands of the expected molecular weight were observed in both cell types. One additional band of 77 kDa was detected by anti-Stim1 in RCC-EPCs. When both Stim1 bands (77 and 100 kDa) were compared to the single band detected at 100 kDa in N-ECFCs, the expression of Stim1 protein became significantly higher in RCC-EPCs (see text for further explanations). Each bar in the upper panel represents the mean±SE of the densitometric analysis of four different experiments. The asterisk indicates p<0.01 (Student's *t*-test).

The expression of Orai1 and Stim1, which mediate SOCE in N-EPCs, was assessed by qRT-PCR analysis of mRNA extracts from RCC-EPCs. The specific primers described in Table 1 have been utilized to examine the expression levels of Orai1-3 and Stim1-2 transcripts. Negative controls were performed by omitting the reverse transcriptase (not shown). The comparison of mRNA levels obtained by qRT-PCR suggested that Orai1 and Stim1, but not Orai2-3 and Stim2, were over-expressed in RCC-EPCs as compared to control cells (Fig. 3A and 3B). A western blot analysis of Orai1 and Stim1 expression was then conducted by employing affinity-purified antibodies. Immunoblots showed a major band of about 33 kDa for Orai1 in both N-EPCs and RCC-EPCs (Fig. 4A). Densitometry of the bands demonstrated that RCC-EPCs exhibited significantly higher levels of Orai1 proteins as compared to control cells (Fig. 4A). As to Stim1 protein, the anti-human Stim1 antibody (Ab) detected a band with an apparent molecular size of 100 kDa in both N-EPCs, as previously shown [27], and an additional band of 77 kDa in RCC-EPCs (Fig. 4B). Based on previous findings, according to which Stim1 is a 77 kDa single-pass transmembrane protein which may undergo post-translational modifications which increase its molecular weight up to 90–100 kDa [37]–[43], these results suggest that RCC-EPCs express two Stim1 variants or forms recognized by the same Ab (see Materials and Methods). This issue was also addressed by performing the immunohistochemical localization of Stim1. Figure S2 shows that Stim1 is homogenously expressed within the cytosol in both N-EPCs (Fig. S4A) and RCC-EPCs (Fig. S4B). There is no detectable difference in the pattern of immunostaining between the two cell types, which is consistent with the ability of the anti-Stim1 Ab to detect both protein variants. Notably, control cells, i.e. cells not exposed to the primary antibody, always exhibited an extremely dim labelling (Fig. S4C). There was no significant difference (p = 0.56) between N-EPCs and RCC-EPCs in the expression levels of the 100 kDa band detected by the anti-Stim1 Ab. However, when both Stim1 bands (77 and 100 kDa) observed in RCC-ECFCs were quantified for densitometric analysis and compared to the single band detected in N-ECFCs (100 kDa), the gene/β-actin ratio became significantly higher (p = 0.0043) in the former cells (Fig. 4B). The protein levels of Stim2 were not probed in this study as it does not regulate SOCE in any cell type hitherto investigated [3], [4]. Overall, these results show that the higher amplitude of SOCE in RCC-EPCs is associated to the up-regulation of Orai1 mRNA and protein and of Stim1 transcripts.

**Table 1:** Demography and cancer history of the treatment naïve, advanced, kidney cancer patient involved.

Subject ID*	Sex	Age (years)	Date of diagnosis	Histology	Fuhrman's nuclear grade	Stage^1^ at the time of sampling	MSKCC^2^ at the time of sampling
M-C	M*	69	30/11/09	PAP II	n.a.*	IV	INTERMEDIATE
F-A	F	60	08/04/10	CC	2	IV	GOOD
M-R	M	68	22/02/11	CC	3	IV	GOOD
C-M	F	65	30/10/97	CC	n.a.	IV	GOOD
DL-M	F	43	11/03/10	PAP II	4	IV	INTERMEDIATE
C-A	M	66	22/08/02	CC	3.4	IV	GOOD
P-E	M	29	07/10/08	CC	3	IV	GOOD
R-L	M	62	02/09/10	CC	3	IV	GOOD
C-L	M	45	30/03/06	CC	3	IV	INTERMEDIATE
F-M	M	63	22/07/10	CC	4	IV	INTERMEDIATE
F-F	M	50	18/11/09	CC	3	IV	GOOD
Z-N	M	59	17/02/10	CC	2	IV	GOOD
U-E	F	80	03/12/07	CC	1.1	IV	INTERMEDIATE
C-Lu	M	37	29/11/09	CC	3	IV	GOOD
DM-T	M	69	10/04/08	PAP II	3	IV	INTERMEDIATE
M-CE	M	55	21/01/10	CC	2	IV	GOOD
G-F	M	45	23/12/10	*HLRCC* *	3.4	IV	GOOD
M-G	M	64	07/01/09	CC	2	IV	GOOD

1 Stage is indicated accrding to the 2002 TNM staging system;.

2 MSKCC Motzer prognostic score for advanced kidney cancer patients; it includes 5 parameters:

- Karnofsky performance status <80%;

- Haemoglobin levels < normal lower limits;

- LDH serum levels >1.5 time the normal upper limit level;

- Corrected calcium >10 mg/dL;

- Absence of prior nephroctomy.

If a patient meets more than 2 criteria the prognosis is POOR, if meets 1–2 criteria is INTERMEDIATE, if meets no criteria is GOOD.

* Abbreviations: ID for “identity”; M for male patients; F for female patients; PAP II for the “Papillary type II” histology; CC for “Clear Cell” histology; HLRCC for “Hereditary leyomiomatosis renal cell carcinoma”; n.a. for “not applicable”.

Albeit Orai1 and Stim1 have been reported to underlie SOCE in N-EPCs [26], it has long been known that tumoral cells may undergo a dramatic remodelling of their Ca^2+^ signalling machinery. In this view, members of the TRPC family of non-selective cation channels, such as TRPC1 and TRPC4, may be involved in store-dependent Ca^2+^ entry in a number of non-excitable cells [44]–[45], including endothelial cells [46]–[47]. Therefore, we sought to monitor the pattern of TRPC channels expression in RCC-EPCs. As depicted in Fig. 3C, the transcripts encoding for TRPC1 were significantly higher as compared to control cells, whereas there was no difference in the levels of TRPC4 mRNA in N-EPCs and RCC-EPCs. Similar to N-EPCs [27], the transcripts encoding for TRPC2, TRPC3, TRPC5, TRPC6, and TRPC7 were absent in RCC-EPCs (data not shown). Immunoblotting revealed that TRPC1 protein was up-regulated in EPCs isolated from patients suffering from RCC (Fig. 4C), while TRPC4 was down-regulated (Fig. 4D) possibly due to post-translational modifications, such as ubiquitination. Collectively, these results suggest that TRPC1 might be involved in SOCE in RCC-EPCs.

### Pharmacological characterization of SOCE in EPCs isolated from healthy donors and RCC-patients

**Figure 5:**
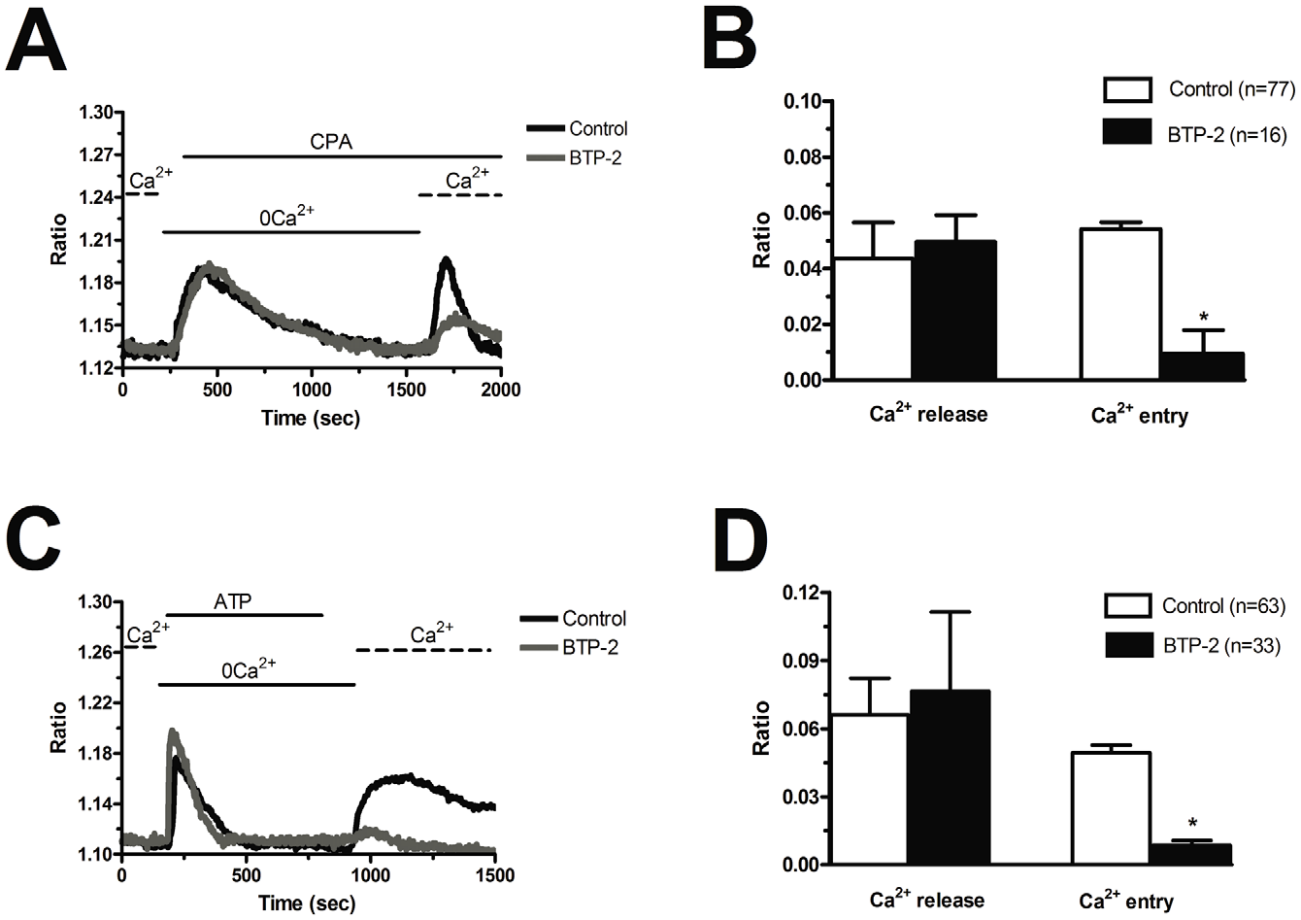
BTP-2 inhibits store-dependent Ca^2+^ entry in endothelial progenitor cells isolated from patients suffering from renal cellular carcinoma. A, CPA-elicited SOCE in the absence (black tracing) and presence (grey tracing) of BTP-2 (20 μM). The cells were pre-incubated with the drug for 20 min before the beginning of the experimental protocol. CPA was administered at 10 μM. B, mean±SE of the amplitude of CPA-induced Ca^2+^ release and CPA-induced SOCE in the absence and presence of BTP-2. The asterisk indicates p<0.05. C, ATP-evoked Ca^2+^ mobilization and SOCE in the presence (black tracing) and absence (grey tracing) of BTP-2 (20 μM, 20 min of pre-treatment). ATP was applied at 100 μM. D, mean±SE of the amplitude of CPA-induced Ca^2+^ release and CPA-induced SOCE in the absence and presence of BTP-2. The asterisk indicates p<0.05.

**Figure 6:**
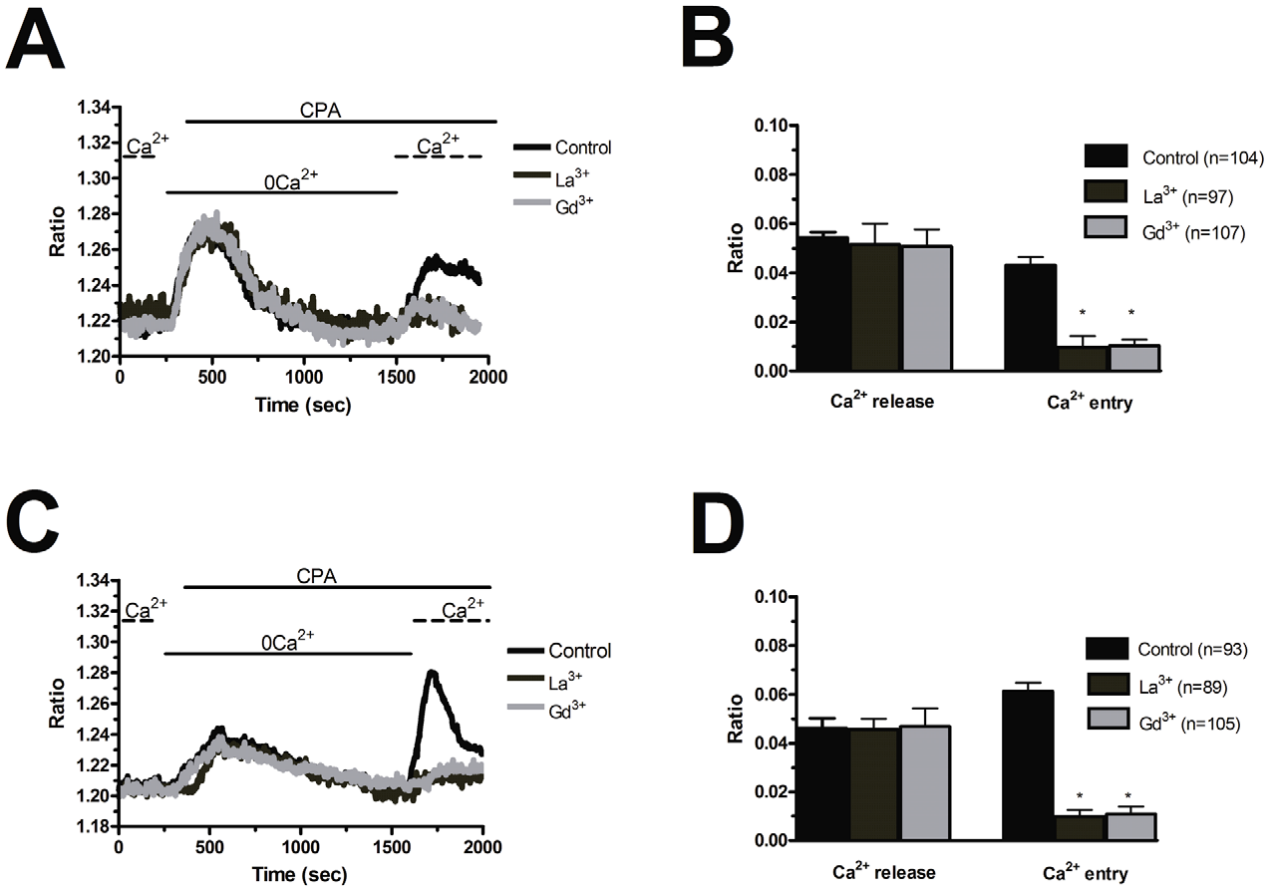
La^3+^ and Gd^3+^ suppress store-operated Ca^2+^ entry evoked by CPA in endothelial progenitor cells. La^3+^ (10 µM) and Gd^3+^ inhibit CPA-induced SOCE in both N-EPCs (A) and RCC-EPCs (C). CPA was applied at 10 µM and the cells were pre-incubated for 20 min with either trivalent cation before store emptying. The traces are representative of the experiments conducted on EPCs from at least three different donors for each condition. Mean±SE of the amplitude of CPA-induced Ca^2+^ release and CPA-induced SOCE in the absence and presence of each trivalent cation in both N-EPCs (C) and RCC-EPCs (D). The asterisk indicates p<0.05.

**Figure 7:**
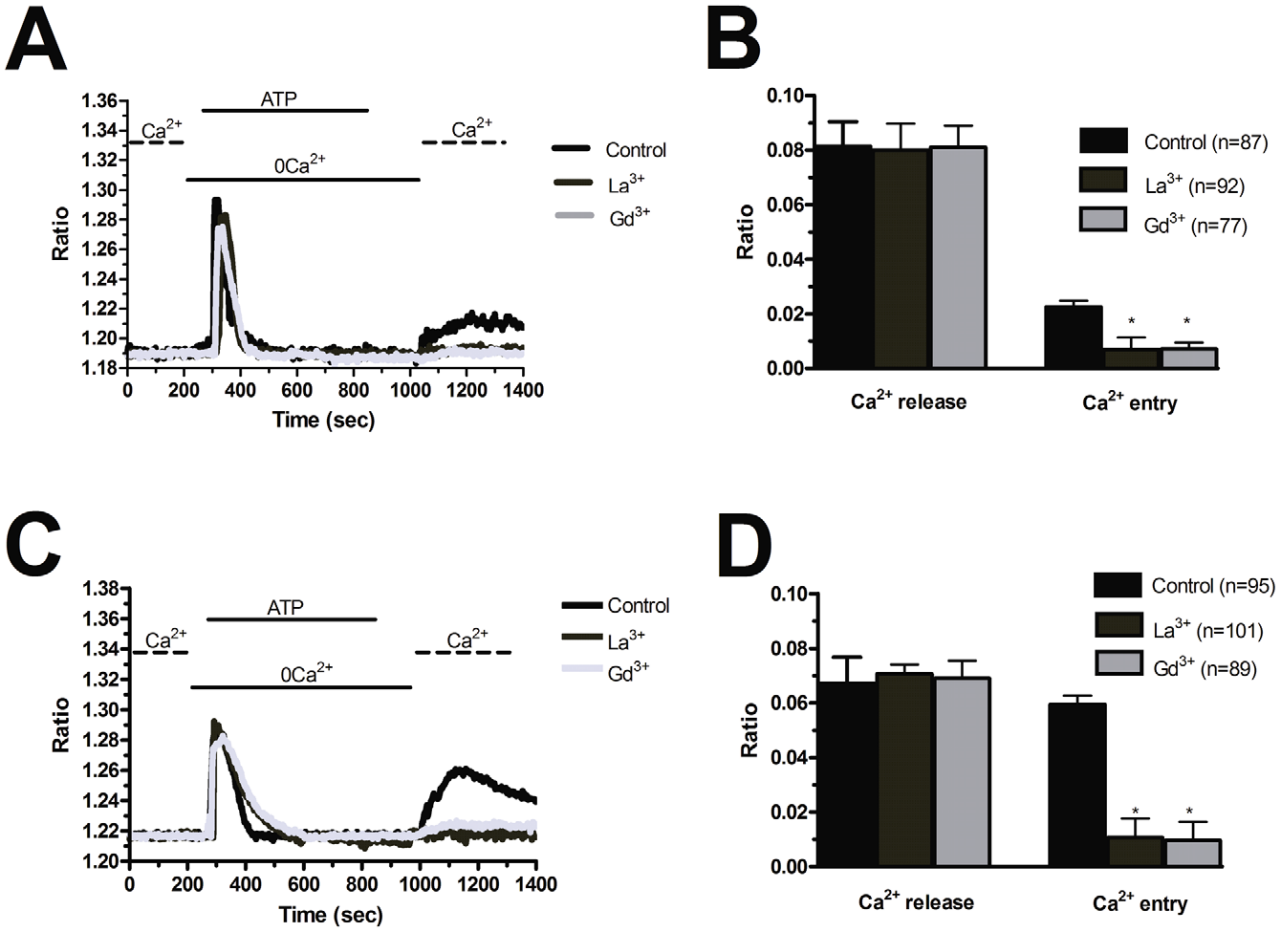
La^3+^ and Gd^3+^ suppress store-operated Ca^2+^ entry stimulated by ATP in endothelial progenitor cells. La^3+^ (10 µM) and Gd^3+^ dampen SOCE triggered by ATP (100 µM) in both N-EPCs (A) and RCC-EPCs (B). The traces are representative of the experiments conducted on EPCs from at least three different donors for each condition. Mean±SE of the amplitude of ATP-induced Ca^2+^ release and ATP-induced SOCE in the absence and presence of each trivalent cation in both N-EPCs (C) and RCC-EPCs (D). The asterisk indicates p<0.05.

**Figure 8:**
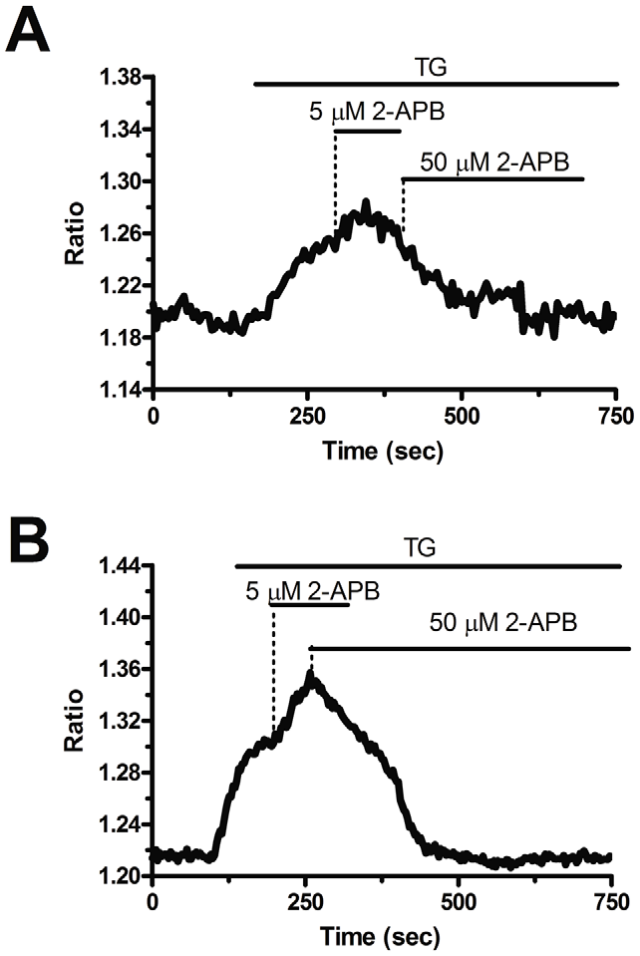
The bi-phasic effect exerted by 2-APB on store-operated Ca^2+^ entry in endothelial colony forming cells. 5 µM 2-APB enhances SOCE induced by thapsigargin (TG), whereas 50 µM inhibits it, in both N-EPCs (A) and RCC-EPCs (B). The traces are representative of, respectively, 51 and 57 cells from at least three independent donors. Thapsigargin was administrated at 2 µM.

Blockers of Ca^2+^ entry might be successfully exploited to affect proangiogenic Ca^2+^ signals in solid tumors [7], [9]. BTP-2 (20 µM), which abrogates store-dependent Ca^2+^ influx and proliferation in N-EPCs [27], [28], selectively reduced SOCE evoked by either CPA (10 µM) (Fig. 5A and Fig. 5B) or ATP (100 µM) (Fig. 5C and Fig. 5D) in RCC-EPCs. The concentration of BTP-2 we employed was suggested by previous studies, which showed that 10–30 µM BTP-2 suppresses SOCE in rat bone marrow-derived colony forming cells-endothelial cells (CFU-ECs) [48], as well as in a variety of cell types [49]–[54]. Consistently, BTP-2 did not affect CPA-induced SOCE at concentrations ≤2 µM in both N-EPCs (Fig. S5A) and RCC-EPCs (Fig. S5B). Therefore, it is conceivable to regard BTP-2 as a selective inhibitor of SOCE in both N-EPCs and RCC-EPCs. In order to confirm that Ca^2+^ entry in response to either CPA or ATP stimulation was operated by store-dependent Ca^2+^ channels, we utilized the trivalent metal cations, La^3+^ and Gd^3+^, which selectively block Orai1-dependent Ca^2+^ entry at 1–10 µM [4], [55]. Ca^2+^ entry triggered by CPA (10 µM) was fully abolished by La^3+^ (10 µM) in both N-EPCs (Fig. 6A and Fig. 6B) and RCC-EPCs (Fig. 6C and Fig. 6D). The same findings were obtained when Ca^2+^ inflow was induced by ATP (100 µM) (Fig. 7A and Fig. 7D). Similarly, the sustained increase in [Ca^2+^]_i_ evoked by CPA and by re-addition of extracellular Ca^2+^ was suppressed by Gd^3+^ (10 µM) in both N-EPCs (Fig. 6A and Fig. 6B) and RCC-EPCs (Fig. 6C and Fig. 6D). The same results were found when Gd^3+^ (10 µM) was probed on ATP-elicited Ca^2+^ inflow in both cell types (Fig. 7A and Fig. 7D). The pharmacological characterization of SOCE in both N-EPCs and RCC-EPCs was further pursued by harnessing the biphasic sensitivity of Stim1/Orai1-mediated Ca^2+^ inrush to 2-APB [4], [55], [56]. It has long been known that 2-APB induces an increase in SOCE evoked by thapsigargin, another well known SERCA inhibitor, at low doses (<5 µM) and transient potentiation followed by blockade at high concentrations (>20 µM). Accordingly, 5 µM 2-APB enhanced thapsigargin-elicited SOCE, whereas 50 µM 2-APB inhibited it, in both N-EPCs (Fig. 8A) and RCC-EPCs (Fig. 8B). Overall, these pharmacological data are consistent with the activation of SOCE by both CPA and ATP in both types of EPCs.

**Figure 9:**
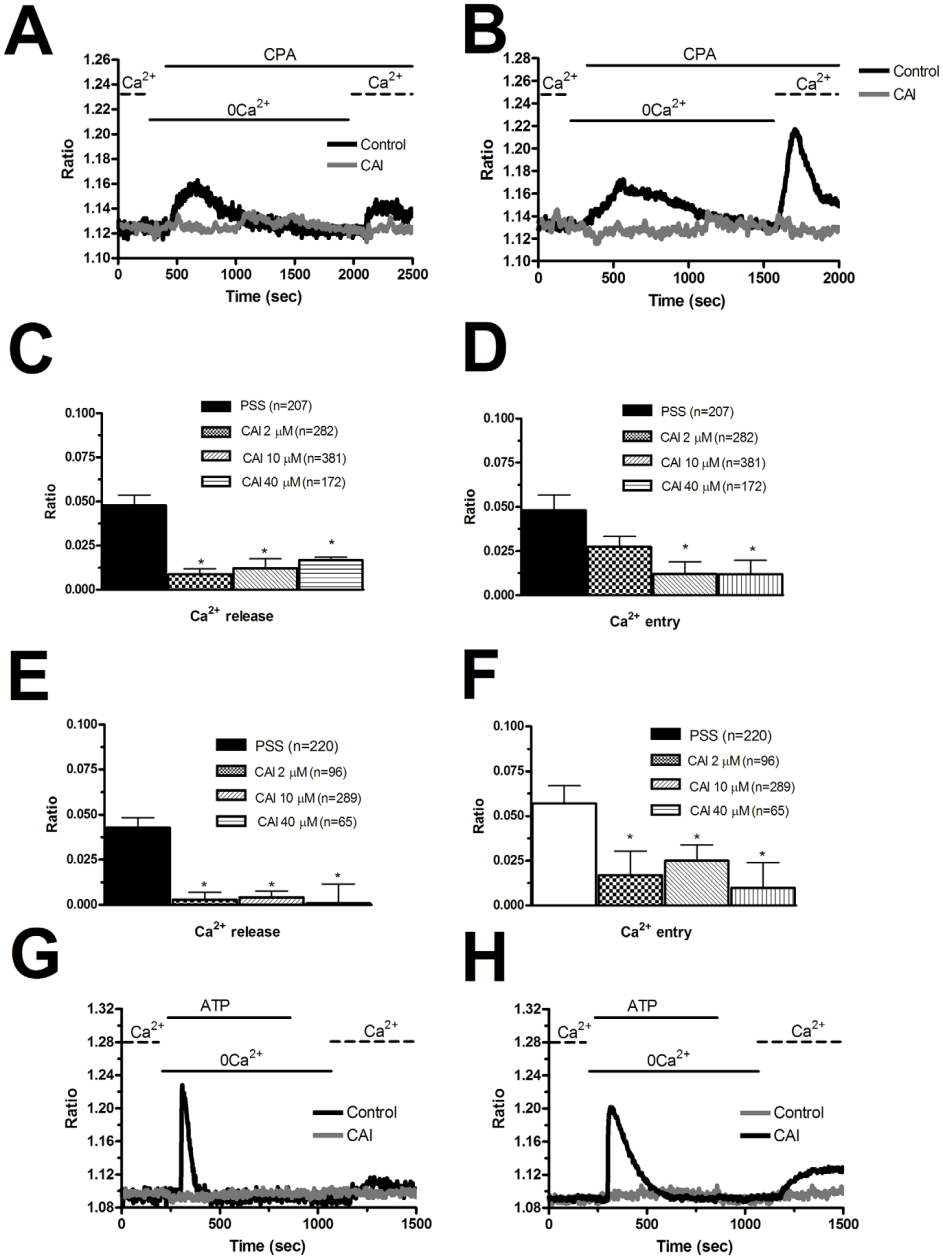
Carboxyamidotriazole suppresses intracellular Ca^2+^ signalling in endothelial progenitor cells. A, 20 min pre-incubation with CAI (10 μM) abolishes the Ca^2+^ response to CPA (10 μM) in N-EPCs. B, 20 min pre-incubation with CAI (10 μM) abolishes the Ca^2+^ response to CPA (10 μM) in RCC-EPCs. C and D, bars indicate the mean±SE of the amplitude of CPA-elicited Ca^2+^ release (C) and SOCE (D) by increasing doses of CAI in N-EPCs. In Panel C, p = 0.00018 for 2 µM, p = 0.00031 for 10 µM, and p = 9.49E-24. In Panel D, p = 0.113 for 2 µM, p = 0.01643 for 10 µM, p = 0.01148 for 40 µM. All these values refer to the magnitude of the control signals. E and F, bars illustrate the mean±SE of the amplitude of CPA-induced Ca^2+^ release (E) and CPA-induced SOCE (F) in RCC-EPCs exposed to increasing concentrations of CAI. In Panel E, p = 0.0016 for 2 µM, p = 0.00025 for 10 µM, and p = 0.02143 for 40 µM. In Panel F, p = 5.30E-26, p = 0–02996 for 10 µM, p = 0.00947 for 40 µM. G, 10 μM CAI prevents both ATP-induced Ca^2+^ release and ATP-elicited SOCE in 70 N-EPCs. H, 10 µM CAI suppresses both ATP-induced Ca^2+^ release and ATP-elicited SOCE in RCC-EPCs.

CAI is a synthetic small molecule inhibitor of non-voltage-gated Ca^2+^ channels that has entered Phase I and II clinical trials both as single cytostatic agent and in combination with cytotoxic therapies (http://clinicaltrials.gov/) [6], [9], [57]. CAI is, however, a non-specific agent that may target cellular pathways other than non-voltage-gated Ca^2+^ channels [7], [55], [57], [58]. Since CAI is currently employed in phase II clinical trials of metastatic RCC [31], [32], we reasoned that it was worth of testing its effect on Ca^2+^ signals in both N-EPCs and RCC-EPCs. Unlike BTP-2, 10 µM CAI inhibited both CPA-elicited Ca^2+^ mobilization and CPA-elicited SOCE (Fig. 9A and 9B). CAI impaired CPA-induced Ca^2+^ signals at 40 µM in N-EPCs (Fig. 9C and 9D) and RCC-EPCs (Fig. 9E and 9F), whereas at 2 µM it only exerted a significant (p<0.05) effect of CPA-dependent SOCE (Fig. 9C–Fig. 9F). Moreover, 10 µM CAI suppressed both the InsP_3_-dependent Ca^2+^ release and the following SOCE when either N-EPCs (Fig. 9G) or RCC-EPCs (Fig. 9H) were stimulated with ATP. Taken as a whole, these data suggest that BTP-2, but not CAI, may be regarded as a *bona fide* blocker of SOCE in RCC-EPCs. The acute addition of CAI (2–10 µM), however, did not trigger any detectable elevation in [Ca^2+^]_i_ in both N-EPCs and RCC-EPCs (Fig. S6). This result rules out the possibility that CAI prevents intracellular Ca^2+^ mobilization by depleting the ER Ca^2+^ reservoir during the period of pre-incubation. That CAI did not interfere with Fura-2, thereby preventing us from recording intracellular Ca^2+^ signals, was demonstrated by the elevation in [Ca^2+^]_i_ evoked by ionomycin (10 µM) in EPCs pre-treated with CAI (not shown).

### SOCE controls RCC-EPC proliferation and tubulogenesis

**Figure 10:**
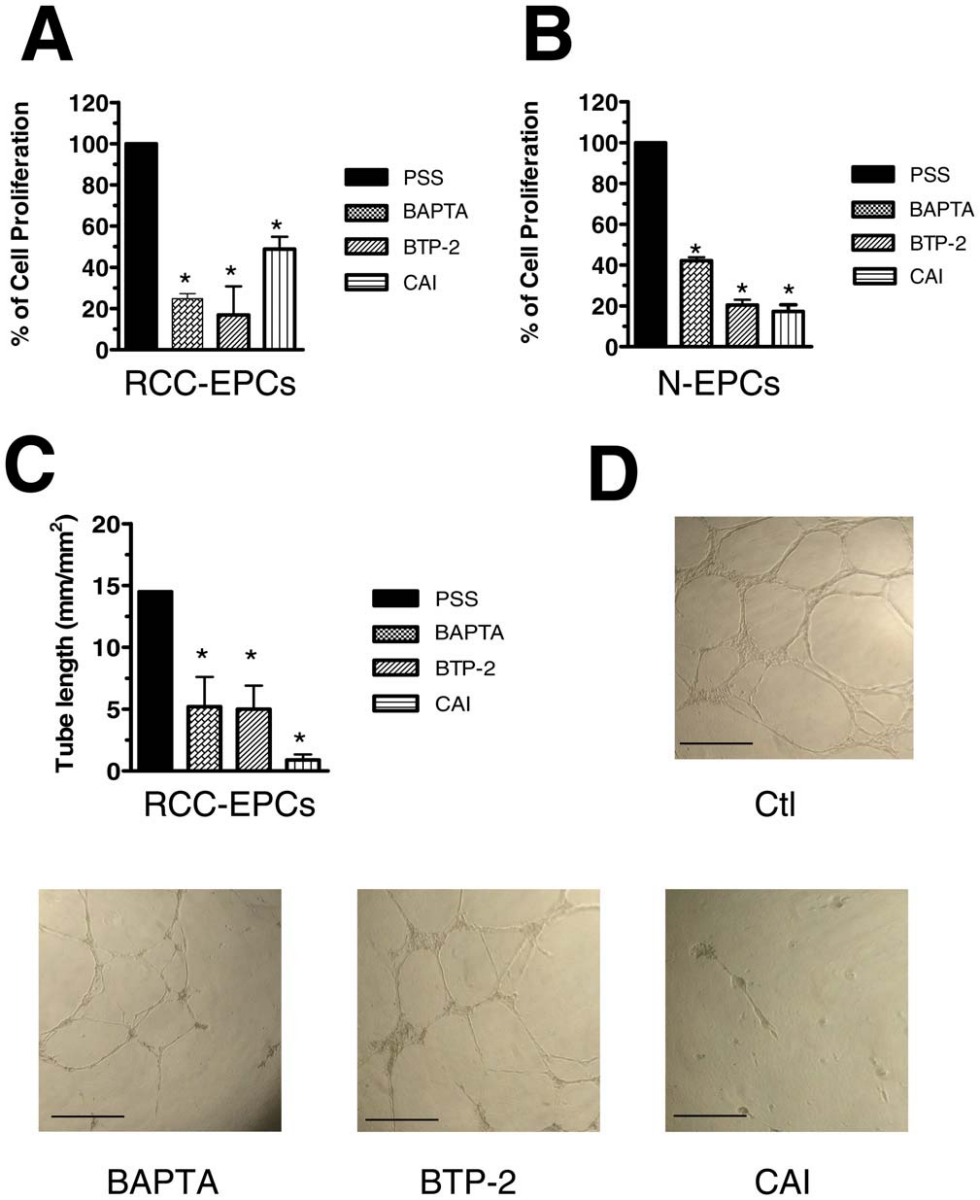
The effect of BAPTA, BTP-2, and carboxyamidotriazole on proliferation and tubulogenesis of endothelial progenitor cells. BAPTA (30 μM), BTP-2 (20 μM), and CAI (10 μM) block the proliferation of both RCC-EPCs (A) and N-EPCs (B). The effect of BTP-2 on N-EPCs has already been shown in [27]. Results are expressed as percentage of growth compared with control (given as 100% growth). C, statistical evaluation of the block exerted by BAPTA (30 μM), BTP-2 (20 μM), and CAI (10 μM) on the assembly into tubulary-like structures of RCC-EPCs. D, digital images of endothelial tubes obtained by bright-field light microscopy 10 hours after plating the cells in Matrigel under control conditions (upper Panel), and in the presence of BAPTA (30 µM), BTP-2 (20 µM), and CAI (10 µM). Cultures were observed up to 24 ours but their appearance did not substantially change after pictures were taken. The bar length is 250 µM.

**Figure 11:**
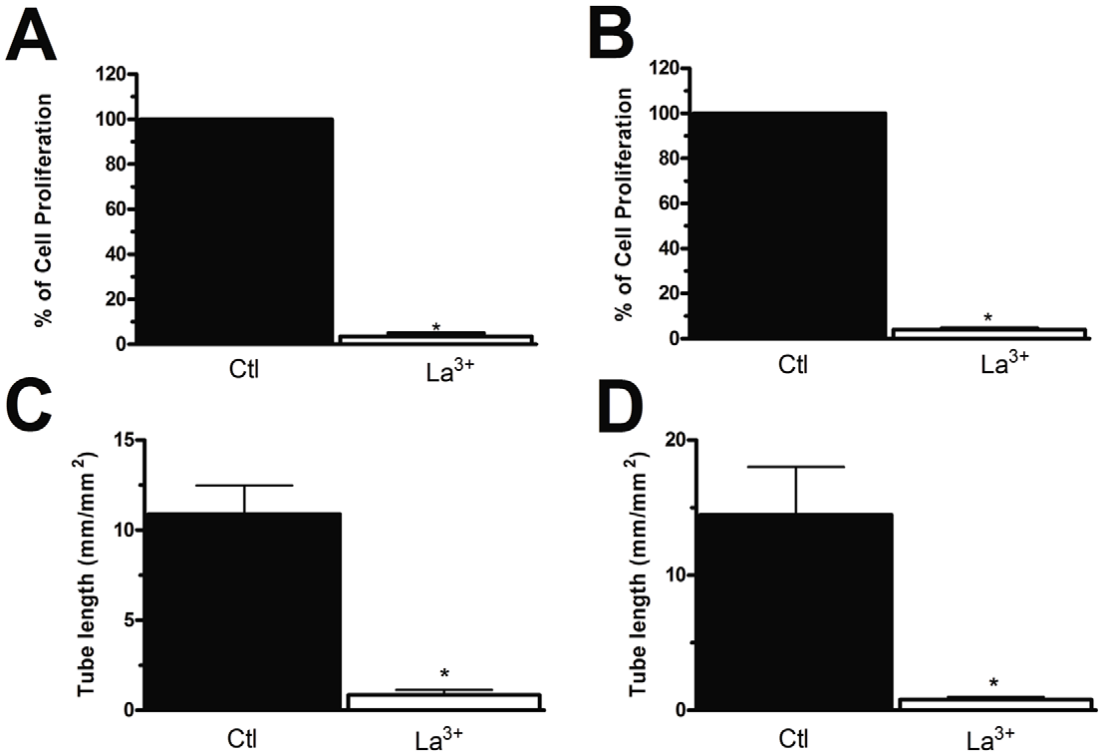
La^3+^ impairs proliferation and tubulogenesis of endothelial progenitor cells. La^3+^ (10 µM) suppresses proliferation in both N-EPCs (A) and RCC-EPCs (B). Results are expressed as percentage of growth compared with control (given as 100% growth). Similarly, La^3+^ (10 µM) interferes with the tubule-formation capacity of both N-EPCs (C) and RCC-EPCs (D).

**Figure 12:**
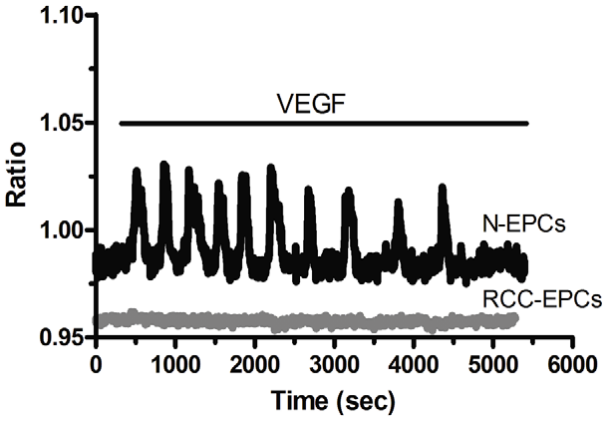
VEGF does not induce Ca^2+^ oscillations in endothelial progenitor cells isolated from patients affected by renal cellular carcinoma. VEGF (10 ng/ml) elicits a burst of intracellular Ca^2+^ waves in N-EPCs (black tracing), but not in RCC-ECFCs (grey tracing). The Ca^2+^ traces are representative of 165 N-EPCs and 148 RCC-EPCs, respectively, harvested from three different donors.

In order to assess whether SOCE controls also RCC-EPC proliferation, we employed the intracellular Ca^2+^ chelator, BAPTA-AM (30 µM), and BTP-2 (20 µM) at doses which have previously been reported to affect cell proliferation [27], [28], [48]–[50], [52], [59]–[66]. Both drugs dramatically affected cell proliferation and prevented the cells from reaching confluence after 3 days of culture (Fig. 10A). BAPTA and BTP-2 also significantly impaired N-EPC growth (Fig. 10B), which is consistent with our previous findings [27], [28]. Similarly, BAPTA (30 µM) and BTP-2 (20 µM) inhibited N-EPC (Fig. S7) and RCC-EPC (Fig. 10C) tube formation when the cells were plated onto matrigel-coated wells. SOCE is, thus, a key determinant of RCC-EPC proliferation and tubulogenesis [27]. Notably, neither BAPTA-AM nor BTP-2 significantly affected cell proliferation when administered at 5 µM and 2 µM, respectively [27] (Fig. S8A and Fig. S8B). Similarly, either drug did not impair tubule formation when employed at these concentrations (Fig. S8C and Fig. S8D). CAI also hindered proliferation and *in vitro* tubulogenesis of both N-EPCs (Fig. 10B and Fig. S7) and RCC-EPCs (Fig. 10A and Fig. 10C) when applied at 10 µM. These effects were not observed when CAI was administrated at 2 µM (Figs. S8A-S8B and Figs. S8C–S8D). However, due to its lack of specificity towards the components of the Ca^2+^ toolkit, we cannot conclude that CAI affects EPCs by selectively targeting SOCE. Therefore, in order to further assess the functional role served by SOCE in RCC-EPCs, cell proliferation and tubule formation were examined in the presence of 10 µM La^3+^. As depicted in Figure 11, such treatment dramatically affected both processes in either cell types. Both of the functional assays described above were conducted in the presence of a growing medium, i.e. EGM-2, containing a number of growth factors, including VEGF. We have recently reported that VEGF causes N-EPCs to undergo both proliferation and tubulogenesis by stimulating intracellular Ca^2+^ oscillations which are maintained over time by SOCE [28]. Surprisingly, VEGF (10 ng/ml) did not elicit any detectable increase in [Ca^2+^]_i_ in these cells, whereas N-EPCs exposed to VEGF at the same dose and diluted from the same stock solution produced the repetitive Ca^2+^ spikes we have previously described (Fig. 12). As a consequence, we sought to assess whether VEGFR-2, the receptor isoform that is selectively coupled to PLC-γ, is down-regulated in RCC-EPCs. VEGFR-2 expression was assessed by flow cytometry, both on ECFC-derived cells obtained from healthy individuals and from RCC patients. No significant difference was observed between the 2 groups: in healthy subjects (n = 4) VEGFR-2 expression ranged from 41 to 63% (median 43%) whereas in patients (n = 4) the median expression was 37.5% (range: 36–44) (P = 0.174). It follows that the physiological agonist responsible for SOCE activation in these cells is unlikely to be VEGF, and should be indentified among the other putative ligands present in the culture medium. Notably, CPA-evoked SOCE was suppressed by genistein (50 µM), a pan-tyrosine kinase receptor blocker, in both N-EPCs (Fig. S9A) and RCC-EPCs (Fig. S9B).

### Molecular characterization of SOCE in RCC-EPCs

**Figure 13:**
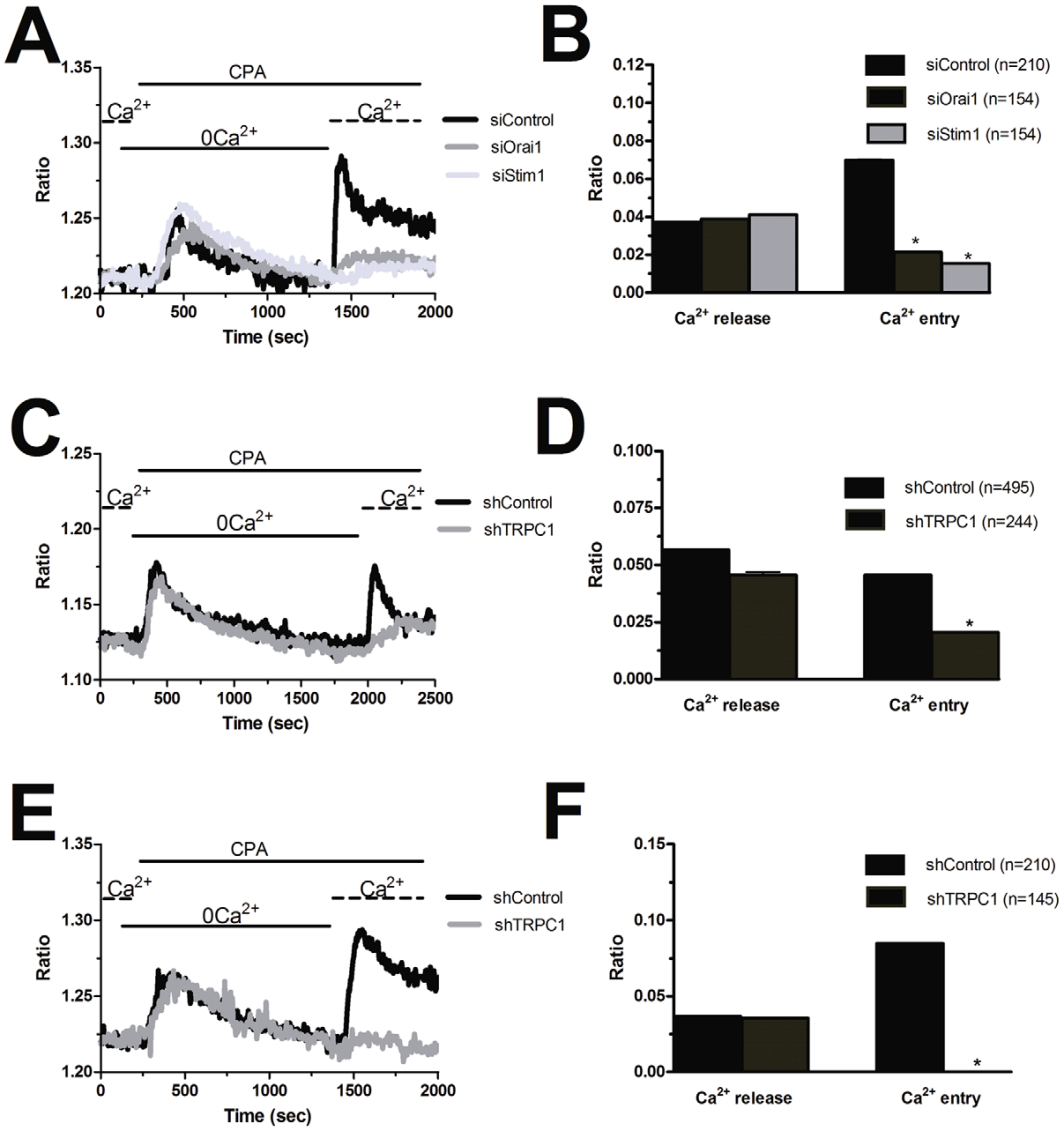
Silencing of Orai1, Stim1, and TRPC1 reduces store-operated Ca^2+^ entry in endothelial progenitor cells. A, SOCE evoked by CPA (10 µM) in RCC-EPCs transfected with a control siRNA (black tracing) or with a specific siRNA sequence devised to knock down either Orai1 (grey tracing) or Stim1 (light gray tracing). B, mean±SE of the amplitude of CPA-induced Ca^2+^ release and CPA-induced SOCE under each condition described in A. The asterisk indicates p<0.05. C, SOCE triggered by CPA (10 µM) in N-EPCs transfected with a scrambled shRNA (black tracing) and with a shRNA selectively targeting TRPC1 (grey tracing). D, mean±SE of the amplitude of CPA-induced Ca^2+^ release and CPA-induced SOCE under each condition described in C. E, SOCE triggered by CPA (10 µM) in RCC-EPCs transfected with a scrambled shRNA (black tracing) and with a shRNA selectively targeting TRPC1 (grey tracing). F, mean±SE of the amplitude of CPA-induced Ca^2+^ release and CPA-induced SOCE under each condition described in E.

As aforementioned, SOCE in N-EPCs is mediated by the physical interaction between Stim1 and Orai1 [26]. Consistent with the higher amplitude of store-dependent Ca^2+^ inflow and with its biphasic sensitivity to 2-APB, both proteins are over-expressed in EPCs harvested from patients affected from RCC. These features, however, are not enough to conclude that Stim1 and Orai1 do mediate SOCE in RCC-EPCs as well. Moreover, TRPC1 mRNA and protein are up-regulated in these cells. Therefore, we sought to unveil the molecular underpinnings of SOCE in RCC-EPCs by first measuring CPA-evoked Ca^2+^ influx in cells transfected with two different siRNA specifically targeting either Stim1 or Orai1, as confirmed at protein level by the immunoblots shown in the Supplementary Material (Fig. S10). As a control, we utilized cells transfected with a scrambled control siRNA. We first focussed on the involvement of Orai1 and Stim1 in RCC-EPCs (Fig. S10A and Fig. S10B). Similar to N-EPCs [27], the knockdown of either Stim1 or Orai1 significantly reduced SOCE in RCC-EPCs, whereas it did not affect the intracellular Ca^2+^ release (Fig. 13A and Fig. 13B). Then, we assessed the role played by TRPC1 in store-dependent Ca^2+^ influx in both N-EPCs and RCC-EPCs by exploiting a specific shRNA sequence against this channel. Western blot analysis demonstrated the effectiveness of TRPC1 expression in both N-EPCs (not shown) and RCC-EPCs (Fig. S10C). The genetic suppression of TRPC1 dramatically inhibited CPA-elicited SOCE in both N-EPCs (Fig. 13C and Fig. 13D) and RCC-EPCs (Fig. 13E and Fig. 13F). Conversely, Ca^2+^ mobilization from intracellular stores was not affected (Figs. 13C–13F). Collectively, these results add two novel pieces of evidence to our understanding of the molecular underpinnings of SOCE in EPCs: 1) TRPC1 participate to store-dependent Ca^2+^ inflow in both N-EPCs and RCC-EPCs along with Stim1 and Orai1; and 2) the higher amplitude of SOCE in EPCs isolated from patients suffering from RCC correlates to the over-expression of Stim1, Orai1, and TRPC1.

## Discussion

SOCE is emerging as a crucial pathway in the control of EPC proliferation, motility, and tubulogenesis [27]–[29]. Concomitantly, emerging evidences indicate that BM-derived EPCs participate to the tumor vascular network in different ways: they favour the formation of primitive tumor endothelium, control tumor growth, and promote the establishment of the (pre)-metastatic niche [18]–[20]. This notion also holds to RCC: this is a typical hypervascular tumor causing an increase mobilization of circulating EPCs, which, in turn, may engraft within tumor neovessels [23]–[25]. Therefore, interfering with the signalling pathways controlling EPC proliferation and tubulogenesis might outline novel molecular targets to treat RCC [15]–[17]. The present investigation provided the first evidence that: 1) SOCE is mediated by Stim1, Orai1, and TRPC1 in EPCs harvested from peripheral blood of RCC patients; 2) the up-regulation of Stim1, Orai1, and TRPC1 is associated to the higher amplitude of SOCE in these cells; and 3) similar to N-EPCs, selective inhibition of SOCE impairs RCC-EPC proliferation and *in vitro* tubulogenesis. These data suggest for the first time that the Ca^2+^ signalling machinery in general, and SOCE in particular, might be exploited to target RCC neovascularisation and improve the outcome of RCC treatments [30].

As in previous studies [27], [33], we used a protocol that consists in restoring Ca^2+^ to the extracellular medium after depletion of endogenous Ca^2+^ stores with either the specific SERCA inhibitor, CPA, or the InsP_3_-synthesizing agonist, ATP. By doing so, we found that SOCE is operative and higher in RCC-EPCs in comparison to N-EPCs. These observations were corroborated by the sub-cellular analysis of CPA-elicited Ca^2+^ inflow in both the sub-membranal space and the mitochondria. The larger amplitude of SOCE in RCC-EPCs was associated to a smaller depletion of the intracellular Ca^2+^ store, as a consequence of the apparent decrease in ER Ca^2+^ content (indicated by the lower Ca^2+^ response to CPA in 0Ca^2+^) and of InsP_3_R down-regulation. This finding is not surprising when considering that the fall in luminal Ca^2+^ content is not linearly correlated to SOCE magnitude [5]. This feature has been well explained by the demonstration of a sub-compartment of the total InsP_3_-sensitive pool which is tightly coupled to SOCE in several cell types [5], including N-EPCs [27]. Therefore, the Ca^2+^ pool that must be emptied for SOCE to be activated is not affected by the global decrease in the ER Ca^2+^ content observed in RCC-EPCs. On the other hand, a substantial increase in Ca^2+^ entry is expected to compensate the reduced basal filling of the intracellular Ca^2+^ reservoir when Ca^2+^ signals play such a crucial role in controlling cell processes as vital as proliferation and tubulogenesis (see below). As recently reviewed in [67], the relative amplitude of SOCE in malignant cells as compared to their non-transformed (i.e. normal) counterparts has been barely assessed owing to the difficulty of obtaining cells from human healthy tissues. SOCE is, however, defective in keratinocytes cultured from patients with neurofibromatosis type 1 [68], in Wilms tumor cells [69], and in chronic myeloid leukemia cells [70]. Conversely, store-dependent Ca^2+^ influx is more robust in malignant B16B6 melanoma cells than in their non-transformed counterparts [71]. The larger magnitude of SOCE in RCC-EPCs was not due to a larger electrochemical gradient for Ca^2+^ entry in these cells, as demonstrated by the experiments conducted in the presence of high-K^+^ extracellular solution. An alternative explanation involves the differential expression of the SOCE machinery in RCC-EPCs. SOCE in human EPCs is mediated by the interplay between Stim1, which serves as the ER Ca^2+^ sensor, and Orai1, which contributes to the channel pore on the plasma membrane [3], [26]. On the other hand, the pore forming subunit of store-dependent channels in rat EPCs is provided by TRPC1 [72], which is informed about the ER Ca^2+^ content by Stim1 [73]. In according with the Ca^2+^ imaging data, Orai1 and TRPC1 were more abundant in RCC-EPCs, as compared to N-EPCs, at both mRNA and protein level. Furthermore, RCC-EPCs displayed an additional Stim1 variant at 77 kDa, which might contribute to enhance SOCE by increasing the number of Orai1 and TRPC1 proteins activated on the plasma membrane by intracellular store depletion. Interestingly, Stim1 has long been known as a single-pass transmembrane protein of 77 kDa which is subject to translational modifications, such as phosphorylation, S-glutathionylation, and glycosylation, that increase its molecular weight up to 90–100 kDa [37]–[42], [74] are targeted to sites other than the epitope of the Santa Cruz Ab we exploited (i.e. the amino acids 441–620 mapping near the COOH-terminal of Stim1). The analysis of Stim1 transcripts reveals an elevation in the mRNA levels in RCC-EPCs: these features suggest that, albeit Stim1 is over-expressed in these cells, not all the translated proteins undergo the same post-translational modifications. This mechanism would explain why two Stim1 isoforms are detected in EPCs isolated from peripheral blood of RCC patients. As a consequence of the 77 kDa band, the global expression of Stim1 protein increased in these cells. In agreement with our observations, *Drosophila* 293T cells display two major bands of Stim1 paralogue, i.e. Stim2, at 105 and 115 kDa as a consequence of their different extent of phosphorylation [75]. It is, however, noteworthy that a doublet of Stim1 bands has recently been detected in a number of BC cell lines, although at a slightly different MW as compared to RCC-EPCs [76]. As recently outlined by Johnstone and colleagues [77], the impact of translational regulation on Stim1 signalling is far from being fully elucidated, but it does not impair its ability to bind to and activate Orai1 and, probably, TRPC1. Future work will, however, be required to unveil the role of the two distinct Stim1 variants in SOCE in RCC-EPCs. Although these data hinted at the involvement of Stim1, Orai1, and TRPC1 in SOCE in RCC-EPCs, the individual knockdown of these proteins was carried out to support this hypothesis at molecular level. Similar to N-EPCs [27], the genetic suppression of either Stim1 or Orai1 severely impaired SOCE in RCC-EPCs. The same result was obtained when both types of EPCs were transfected with shRNAs selectively directed against TRPC1. Therefore, these data concur with those reported by Beech's Group [27], and further extend our knowledge on the molecular composition of SOCE in EPCs by demonstrating TRPC1 involvement in both N-EPCs and RCC-EPCs isolated from human blood. A number of models have been put forward to describe the contribution of Stim1, Orai1, and TRPC1 to store-dependent Ca^2+^ inflow. According to the first scenario, Orai1 and TRPC1 form distinct channels on the plasma membrane that are both activated by Stim1 following depletion of the intracellular Ca^2+^ reservoir. In this case, Orai1 and TRPC1 would mediate Ca^2+^-selective and non-selective cation currents, respectively. The second hypothesis suggests the assembly of a supramolecular ternary complex involving all of the three proteins upon emptying of the ER Ca^2+^ pool [44], [45]. It is worth of noting that a recent investigation conducted on mice pulmonary artery endothelial cells demonstrated that Orai1 may associate to a TRPC1/TRPC1 heterocomplex and convert it into a Ca^2+^-selective channel after intracellular Ca^2+^ release [46].

An increase in Orai1, but not Stim1, levels has also been reported in several breast cancer cell (BCC) lines [11], while the loss of Stim1 protein prevents SOCE activation in Wilms tumor cells [69]. Similarly, TRPC1 is more abundant in human breast ductal adenocarcinoma (hBDA), where it has been correlated to proliferative parameters, but not Ca2^+^ entry [78]. In addition to Stim1, Orai1, and TRPC1, the lower ER Ca^2+^ content and slower decay to the baseline of ATP- and CPA-induced Ca^2+^ release suggest that other components of the Ca^2+^ toolkit are aberrantly expressed in RCC-EPCs. Additional experiments are required to assess the intracellular levels of a number of proteins involved in the ER Ca^2+^ cycle and in Ca^2+^ extrusion across the plasma membrane [1]. The up-regulation of Orai1, as well as the decrease in InsP_3_R transcripts, and the appearance of an additional Stim1 variant suggest that RCC-EPCs display a different gene expression profile as compared to N-EPCs. This feature concurs with the notion that tumor ECs (TECs) exhibit tumor-specific sets of up-regulated genes and chromosomal aberrations [79], [80]. For instance, TECs from human RCC over-express genes that promote endothelial proliferation, survival, motility, and cell adhesion [79]. In agreement with our results, TECs harvested from breast cancer generate abnormal Ca^2+^ signals when exposed to arachidonic acid (AA), a pro-angiogenic intracellular messenger [81]–[82]. Normal ECs may undergo genetic and epigenetic modifications by receiving DNA directly from the tumor through apoptotic bodies, or following mRNA and microRNA transfer by microvesicles [80]. This novel mode of intercellular communication occurs both locally and systemically. It is, therefore, conceivable that either bone marrow resident or circulating EPCs remodel their Ca^2+^ toolkit in response to instructive signals emitted from RCC microenvironment [80].

We have previously shown that store-dependent Ca^2+^ influx and proliferation in N-EPCs is sensitive to BTP-2 [27], a pyrazole derivative that selectively affects SOCE and its downstream targets in T cells peripheral lymphocytes and mast cells [4]. However, the requirement for Ca^2+^ entry to drive RCC-EPC proliferation and tubulogenesis could not be given for granted. Indeed, it has long been known that cancer cells may keep on proliferating even in a Ca^2+^-poor medium, a process that has been termed “habituation” to low extracellular Ca2^+^ [9], [12], [13]. Although there is no evidence that RCC-EPCs derive from the neoplastic clone, so that they cannot be regarded as tumoral cells, it was necessary to assess their need for SOCE to undergo angiogenesis. In the present investigation, we found that BTP-2 blocked both CPA-elicited and ATP-elicited Ca^2+^ entry, but not intracellular Ca^2+^ release, in RCC-EPCs. The same results were obtained when both types of EPCs were stimulated in the presence of low micromolar concentrations of either La^3+^ or Gd^3+^, which selectively target Orai1-mediated Ca^2+^ inflow. Therefore, BTP-2 and La^3+^ could be exploited to assess the role played by SOCE in two key processes of EPC-mediated tumor vascularisation, such as cell growth and tubulogenesis. BTP-2 and La^3+^, as well as the intracellular Ca^2+^ buffer, BAPTA, blocked RCC-EPC proliferation. The involvement of SOCE in the control of cell growth has also been described in a variety of malignancies [52], [83], [84]. As extensively reviewed elsewhere [2], [7], [10], [30], recruitment of the CaM/CaMK pathway by SOCE is key to cell cycle progression through G_1_ and mitosis by regulating the activation of several cyclin-dependent kinases (cdk), such as cdk2 and cdk4, and of calcineurin. BTP-2 and La^3+^ were also found to impair *in vitro* tubulogenesis in both N- and RCC-EPCs. This result concurs with the notion that EC adhesion to substrate, spreading, motility and establishment of a patent lumen require extracellular Ca^2+^ entry [7], [57]. This process is mediated by Ca^2+^-sensitive decoders, such as CaMKII and the neutral protease calpain, that cleaves several focal adhesions proteins at the trailing edge [85]. Consistent with our data, SOCE modulates cell motility and invasion in a number of tumor cells [86]–[88]. Unlike BTP-2 and trivalent cations, CAI exerted a non-specific inhibitory effect on intracellular Ca^2+^ signalling in EPCs. First, pre-treatment with CAI abolished both phases (i.e. intracellular Ca^2+^ release and SOCE) of the Ca^2+^ response to CPA. CAI, however, did not cause any detectable increase in [Ca^2+^]_i_ when acutely administered to EPCs. Thus CAI did not deplete the intracellular Ca^2+^ reservoir during the pre-incubation period. Moreover, CAI did not interfere with Fura-2 fluorescence. CPA-evoked Ca^2+^ efflux from ER is mediated by yet to be identified leakage channels [1]. In this light, our results suggest that CAI may block these pathways, thereby preventing the fall in ER Ca^2+^ levels consequent to SERCA inhibition by CPA. Blockage of SOCE in cells pre-treated with CAI might be due to the upstream inhibition of the signal transduction pathway leading to Orai1 activation. Second, CAI suppressed both InsP_3_-dependent Ca^2+^ release and SOCE in ATP-stimulated EPCs. Once again, CAI might act by preventing the InsP_3_-mediated drop in ER Ca^2+^ levels that must be sensed by Stim1 to stimulate Orai1. The strong impairment of Ca^2+^ signalling by CAI leads to a significant reduction in the extent of cell growth and tubulogenesis in both N-EPCs and RCC-EPCs. Unlike BTP-2 and La^3+^, which selectively affects SOCE, we cannot rule out that CAI inhibits EPC by affecting additional signal transduction pathways. It is, however, intriguing that lower doses (i.e. 2 µM) of CAI, which are ineffective on SOCE, do not block but not EPC proliferation and tubulogenesis. Lack of specificity is a hallmark of CAI, which has already been shown to block InsP_3_ synthesis in mature ECs [58] and InsP_3_-dependent Ca^2+^ mobilization in human head and neck squamous cell carcinoma [89]. Moreover, CAI inhibits the mitochondrial Ca^2+^ uniporter [90], AA-induced Ca^2+^ inflow [81], and SOCE [91]. A number of severe toxicities, such as neuropathies and gastrointestinal disorders, have been described during Phase II clinical trials of patients with advanced RCC and refractory to immunotherapy [31], [32]. These untoward side-effects, which are absent in subjects treated with drugs selectively targeting Ca^2+^-permeable channels [6], might depend on the unspecific effects of CAI on Ca^2+^ signals. Albeit our data clearly indicate that SOCE drives proliferation and tubulogenesis in RCC-EPCs, the physiological agonist of this pathway remains to be elucidated. We have previously shown that VEGF recruits SOCE to sustain the repetitive Ca^2+^ spikes that underlie its pro-angiogenic effect on N-EPCs. Nevertheless, VEGF is ineffective at stimulating detectable Ca^2+^ signals in RCC-EPCs despite the fact that VEGFR-2 is not down-regulated as compared to control cells. This result might be explained by the reduction in InsP_3_R levels in these cells. The amount of InsP_3_ produced by VEGFR-2 might not be sufficient to stimulate Ca^2+^ release and engage SOCE. The culture medium employed to stimulated cell proliferation and tubulogenesis, i.e. EGM-2, contains a number of additional growth factors and cytokines, such as EGF, bFGF and bovine foetal serum, that might stimulate RCC-EPC by enlisting SOCE [7], [30], [92].

In conclusion, this study demonstrated for the first time that SOCE is up-regulated and controls proliferation and tubulogenesis in RCC-EPCs. The higher amplitude of SOCE in these cells is associated to the over-expression of its molecular players, namely Stim1, Orai1, and TRPC1. Targeted therapy based on tyrosine kinase inhibitors does not lead to a significant increase in progression-free survival and survival rates of patients suffering from RCC [15]. Moreover, these treatments may lead to bothersome side-effects which severely affect the quality of life of the patients [16]. Ca^2+^-permeable channels are regarded as novel molecular targets to exploit in the fight against the cancer [6], [7], [9]. SOCE might, therefore, be harnessed to devise alternative strategies to affect highly vasculogenic tumors, such as RCC [30]. In this view, Stim1, Orai1, and TRPC1 stand out as very promising molecular identities to hit to adverse tumor neovascularization. This feature gains much more relevance when considering that VEGF fails to elicit intracellular Ca^2+^ signals in RCC-EPCs. This finding does not rule out the possibility that VEGFR-2 triggers alternative signalling pathways in these cells, such as the phosphoinositide 3-kinase (PI3K)/Akt and the p38 mitogen-activated protein kinase (p38 MAPK) pathways. However, it provides the first molecular explanation for the modest increase in the overall survival and progression-free survival of RCC patients treated with a humanized anti-VEGF neutralizing monoclonal antibody, such as bevacizumab, and the tyrosine kinase inhibitors, sorafenib and sunitinib. These drugs might, indeed, be directed against a target, i.e. VEGFR-2, which is uncoupled from its intracellular signaling cascade in one of the most important cell populations, i.e. EPCs, which contribute to establish the vascular network within the tumor. In this view, the discovery of the role served by Stim1, Orai1, and TRPC1 in driving RCC-EPC proliferation and tubulogenesis might be helpful in devising alternative strategies, such as the local administration of siRNA targeting the molecular components of SOCE or the intratumoral delivery of selective SOCE blockers via the polymer implants that have been recently described [30].

## Experimental Procedures

### Isolation and cultivation of endothelial colony forming cells

Blood samples (40 mL) were obtained from healthy human volunteers aged from 22 to 48 years old (n = 12) and from RCC patients (n = 18). Demographic and clinical characteristics of patients are summarized in Table 2. The Institutional Review Board at “Istituto di Ricovero e Cura a Carattere Scientifico Policlinico San Matteo Foundation” in Pavia approved all protocols and specifically approved this study. Informed written consent was obtained according to the Declaration of Helsinki. We focussed on the so-called endothelial colony forming cells (ECFCs) [27], [28], a subgroup of EPCs which are found in the CD34^+^ CD45^−^ fraction of circulating mononuclear cells, exhibit robust proliferative potential and form capillary-like structures *in vitro* [93], [94]. To isolate ECFCs, mononuclear cells (MNCs) were separated from peripheral blood (PB) by density gradient centrifugation on lymphocyte separation medium for 30 min at 400 g and washed twice in EBM-2 with 2% FCS. A median of 36×10^6^ MNCs (range 18–66) were plated on collagen-coated culture dishes (BD Biosciences) in the presence of the endothelial cell growth medium EGM-2 MV Bullet Kit (Lonza) containing endothelial basal medium (EBM-2), 5% foetal bovine serum, recombinant human (rh) EGF, rhVEGF, rhFGF-B, rhIGF-1, ascorbic acid and heparin, and maintained at 37°C in 5% CO_2_ and humidified atmosphere. Discard of non-adherent cells was performed after 2 days; thereafter medium was changed three times a week. The outgrowth of ECs from adherent MNCs was characterized by the formation of a cluster of cobblestone-appearing cells [27], [94]. That ECFC-derived colonies belonged to endothelial lineage was confirmed as described in Sánchez-Hernández et al. [27] and Piaggio et al. [95]. In more detail, EPC-derived colonies were stained with anti-CD31, anti-CD105, anti-CD144, anti-CD146, anti-vWf, anti-CD45, and anti-CD14 monoclonal antibodies (see Table S1) and by assessment of capillary-like network formation in an *in vitro* Matrigel assay (see Figure 9 and Figure 10 and [27]). VEGFR-2 expression was also evaluated. 1×10^5^ ECFC-derived cells, obtained from sub-confluent cultures, were incubated for 30 min at 4 C with a biotin-conjugated anti-VEGFR2 monoclonal antibody (Sigma), revealed by 5 mcl of 1∶5 diluted PerCp-streptavidin (Becton Dickinson). An appropriate isotype was used as control. Cells were acquired by a FACS Calibur flow cytometer and analysed by the CEllQuest software (BD Biosciences).

**Table 2:** Primer sequences used for real time reverse transcription/polymerase chain reaction of Orai1-3, Stim1-2, TRPC1-7.

Gene	Primer sequences		Size (bp)	Accession number
Orai1	Forward	5′-AGTTACTCCGAGGTGATGAG-3′	257	NM_032790.3
	Reverse	5′-ATGCAGGTGCTGATCATGAG-3′		
Orai2	Forward	5′-CCATAAGGGCATGGATTACC-3′	334	NM_001126340.1variant 1
	Reverse	5′-CAGGTTGTGGATGTTGCTCA-3′		NM_032831.2variant 2
Orai3	Forward	5′-CCAAGCTCAAAGCTTCCAGCC-3′	159	NM_152288.2
	Reverse	5′-CAAAGAGGTGCACAGCCACCA-3′		
Stim1	Forward	5′-CCTCAGTATGAGGAGACCTT-3′	347	NM_003156.3
	Reverse	5′-TCCTGAAGGTCATGCAGACT-3′		
Stim2	Forward	5′-AAACACAGCCATCTGCACAG-3′	186	NM_020860.2
	Reverse	5′-GGGAAGTGTCGTTCCTTTGA-3′		
TRPC1	Forward	5′-ATCCTACACTGGTGGCAGAA-3′	307	NM_003304.4
	Reverse	5′-AACAAAGCAAAGCAGGTGCC-3′		
TRPC3	Forward	5′-GGAGATCTGGAATCAGCAGA-3′	336	NM_001130698.1 variant 1
	Reverse	5′-AAGCAGACCCAGGAAGATGA-3′		NM_003305.2 variant 2
TRPC4	Forward	5′-ACCTGGGACCTCTGCAAATA-3′	300	NM_016179.2 variant alpha
	Reverse	5′-ACATGGTGGCACCAACAAAC-3′		NM_001135955.1 variant beta
				NM_001135956.1 variant gamma
				NM_001135957.1 variant delta
				NM_003306.1 variant epsilon
				NM_001135958.1 variant zeta
TRPC5	Forward	5′-GAGATGACCACAGTGAAGAG-3′	221	NM_012471.2
	Reverse	5′-AGACAGCATGGGAAACAGGA-3′		
TRPC6	Forward	5′-AGCTGTTCCAGGGCCATAAA-3′	341	NM_004621.5
	Reverse	5′-AAGGAGTTCATAGCGGAGAC-3′		
TRPC7	Forward	5′-CACTTGTGGAACCTGCTAGA-3′	387	NM_020389.1
	Reverse	5′-CATCCCAATCATGAAGGCCA-3′		
β-actin	Hs_ACTB_1_SG, QuantiTect Primer Assay QT00095431, Qiagen		146	NM_001101

For our experiments, we have mainly used endothelial cells obtained from early passage ECFC (P1–3, which roughly encompasses a 15–18 day period) with the purpose to avoid (or maximally reduce) any potential bias due to cell differentiation. However, in order to make sure that the phenotype of the cells did not change throughout the experiments, in preliminary experiments we tested the immunophenotype of both normal ECFCs and ECFCs harvested from RCC patients at different passages and we found no differences, as shown in the Table S1 where the median values of cells expressing the specific antigens are reported. We also tested whether functional differences occurred when early (P2) and late (P6) passage-ECFCs were used by testing the in vitro capacity of capillary network formation in a Matrigel assay and found no differences between early and late passage ECFC-derived cells.

### Solutions

Physiological salt solution (PSS) had the following composition (in mM): 150 NaCl, 6 KCl, 1.5 CaCl_2_, 1 MgCl_2_, 10 Glucose, 10 Hepes. In Ca^2+^-free solution (0Ca^2+^), Ca^2+^ was substituted with 2 mM NaCl, and 0.5 mM EGTA was added. Solutions were titrated to pH 7.4 with NaOH. The high-K^+^ extracellular solution was prepared by replacing 100 mM NaCl with an equimolar amount of KCl. The solution was then titrated to pH 7.4 with KOH. The osmolality of the extracellular solution, as measured with an osmometer (Wescor 5500, Logan, UT), was 300–310 mmol/kg.

### [Ca^2+^]_i_ measurements

EPCs were loaded with 4 µM fura-2 acetoxymethyl ester (fura-2/AM; 1 mM stock in dimethyl sulfoxide) in PSS for 1 hour min at room temperature. After washing in PSS, the coverslip was fixed to the bottom of a Petri dish and the cells observed by an upright epifluorescence Axiolab microscope (Carl Zeiss, Oberkochen, Germany), usually equipped with a Zeiss ×40 Achroplan objective (water-immersion, 2.0 mm working distance, 0.9 numerical aperture). EPCs were excited alternately at 340 and 380 nm, and the emitted light was detected at 510 nm. A first neutral density filter (1 or 0.3 optical density) reduced the overall intensity of the excitation light and a second neutral density filter (optical density = 0.3) was coupled to the 380 nm filter to approach the intensity of the 340 nm light. A round diaphragm was used to increase the contrast. The excitation filters were mounted on a filter wheel (Lambda 10, Sutter Instrument, Novato, CA, USA). Custom software, working in the LINUX environment, was used to drive the camera (Extended-ISIS Camera, Photonic Science, Millham, UK) and the filter wheel, and to measure and plot on-line the fluorescence from 10–15 rectangular “regions of interest” (ROI) enclosing 10–15 single cells. Each ROI was identified by a number. Since cell borders were not clearly identifiable, a ROI may not include the whole EPC or may include part of an adjacent EPC. Adjacent ROIs never superimposed. [Ca^2+^]_i_ was monitored by measuring, for each ROI, the ratio of the mean fluorescence emitted at 510 nm when exciting alternatively at 340 and 380 nm (shortly termed “ratio”). An increase in [Ca^2+^]_i_ causes an increase in the ratio [27]. Ratio measurements were performed and plotted on-line every 3 s. In a number of experiments, background fluorescence, when exciting at 340 nm and 380 nm, respectively, was evaluated in regions void of cells and subtracted on line. The experiments were performed at room temperature (22°C). All the data have been collected from EPCs isolated from the peripheral blood of at least three healthy volunteers or three RCC patients. The amplitude of the peak Ca^2+^ response to either CPA or ATP was measured as the difference between the ratio at the peak and the mean ratio of 1 min baseline before the peak. SOCE amplitude was measured by averaging 60 sec of signal at the peak and subtracting to this value the mean ratio of 1 min baseline recorded before Ca^2+^-readdition. Pooled data are given as mean±SE and statistical significance (*P*<0.05) was evaluated by the Student's *t*-test for unpaired observations.

### Lentiviral transduction with pmAEQ and mitAEQ and aequorin Ca^2+^ measurement

Cloning of the lentiviral construct expressing aequorin (AEQ) N-terminally linked to the synaptic-associated protein 25 (SNAP-25) targeted to the sub-plasma membrane space (pLV-pmAEQ) and of the construct targeted to the mitochondrial lumen using COXVIII cleavable leader sequence (pLV-mitAEQ-IRES-EGFP) were described elsewhere [96], [97]. The lentiviral particles were produced as described in Lazzari et al. [97] with modifications. Briefly, EPCs were transfected with pMDLg/pRRE, pMD2.VSVG, pRSV-Rev and pLV-pmAEQ (or pLV-mitAEQ-IRES-EGFP), by means of the calcium-phosphate transfection method. After 10 h, the transfection medium was replaced with fresh culture medium, and cells were grown for further 72 h. The medium was then collected, filtered through a 0.45 µm PES filter (Millipore Corporation, Bedford, MA, USA) and viral particles were precipitated by PEG solution (8% PEG8000, 100 mM NaCl, 0.4 mM TrisHCl, pH 7.2). Finally pellet was resuspended in 0.5 mL of phosphate buffered saline (PBS), aliquoted, and stored at 80°C until use. Lentiviral stock was tittered by infecting EPC cells by serial dilutions and anti HA-immunocytochemistry and EGFP fluorescence. The dilutions that resulted >70–80% of infected cells were used for AEQ experiments. For AEQ Ca^2+^ measurement, RCC-EPCs and N-EPC cells were spotted onto fibronectin-coated 13 mm coverslips in a 24 well plate (Costar) at a density 2–3×10^3^ cells per spot. 24 ours after plating the cells were infected with pmAEQ and mitAEQ expressing lentivirus for 48–72 ours. Then, the cells were reconstituted with coelenterazine, placed on a thermostated chamber of a custom built luminometer (CAIRN research, Kent, UK). Emitted light was converted in Ca^2+^ concentrations offline using a previously described algorithm [98]. All measurements were carried out at 37°C.

### RNA isolation and real time RT-PCR (qRT-PCR)

Total RNA was extracted from the EPCs using the QIAzol Lysis Reagent (QIAGEN, Italy). Single cDNA was synthesized from RNA (1 μg) using random hexamers and M-MLV Reverse Transcriptase (Invitrogen S.R.L., Italy). Reverse transcription was always performed in the presence or absence (negative control) of the reverse transcriptase enzyme. qRT-PCR was performed in triplicate using 1 μg cDNA and specific primers (intron-spanning primers; Table 2 and Table S2), as previously described elsewhere [27]. MESA GREEN qPCR MasterMix Plus (Eurogentec, Liege Science Park, Belgium) was used according to the manufacturer instruction and qRT-PCR performed using Rotor Gene 6000 (Corbett, Concorde, NSW, Australia). The conditions were as follows: initial denaturation at 95°C for 5 min; 40 cycles of denaturation at 95°C for 30 sec; annealing at 58°C for 30 sec, and elongation at 72°C for 40 sec. The qRT-PCR reactions were normalized using β-actin as housekeeping gene. Melting curves were generated to detect the melting temperatures of specific products immediately after the PCR run. The triplicate threshold cycles (Ct) values for each sample were averaged resulting in mean Ct values for both the gene of interest and the housekeeping gene β-actin. Relative mRNA levels were determined by comparative quantitation (Corbett) and the results expressed as fold change. The sequences of the bands were checked by using the Big dye terminator cycle sequencing kit (Applied Biosystem, PE, USA). PCR products were also separated with agarose gel electrophoresis, stained with ethidium bromide, and acquired with the Image Master VDS (Amersham Biosciences Europe, Italy). The molecular weight of the PCR products was compared to the DNA molecular weight marker VIII (Roche Molecular Biochemicals, Italy).

### Sample preparation and immunoblotting

N-EPCs and RCC-EPCs were homogenized by using a Dounce homogenizer in a solution containing: 250 mM Sucrose, 1 mM EDTA, 10 mM Tris-HCl, pH 7.6, 0.1 mg/ml PMSF, 100 mM β-mercaptoethanol and Protease Inhibitor Cocktail (P8340, Sigma, USA). The homogenates were solubilized in Laemmli buffer [27] and 20 µg proteins were separated on 10% SDS-polyacrilamide gel electrophoresis and transferred to the Hybond-P PVDF Membrane (GE Healthcare, Italy) by electroelution. After 1 h blocking with Tris buffered saline (TBS) containing 3% BSA and 0.1% Tween (blocking solution), the membranes were incubated for 3 h at room temperature with affinity purified antibodies diluted 1∶200 in the TBS and 0.1% Tween. Anti-Stim1 (sc-166840) was from Santa Cruz Biotechnology Inc. (Santa Cruz, CA, USA), and anti-Orai1 was from Alomone Labs Ltd. (Jerusalem, Israel).

The membranes were washed and incubated for 1 h with peroxidase-conjugated mouse, or rabbit IgG (1∶120000 in blocking solution) (QED Bioscience, Inc., U.S.A.). The bands were detected with the ECL™ Advance western blotting detection system (GE Healthcare Europe GmbH, Italy). Control experiments were performed as described in Sánchez-Hernández et al. [27]. BenchMark pre-stained protein ladders (Invitrogen, Carlsbad, CA, USA) were used to estimate the molecular weights. The ChemiBlot™ Molecular Weight Markers were used to accurately estimate the molecular weight and as a positive control for the immunoblot (Chemicon International, Inc., CA, USA). Blots were acquired with the Image Master VDS (Amersham Biosciences Europe, Italy). Densitometric analysis of the bands was performed by the Total Lab V 1.11 computer program (Amersham) and the results were expressed as a percentage of the gene/β-actin densitometric ratio. Blots were stripped [99] and re-probed with anti β-actin rabbit antibody as loading control (Rockland Immunochemicals for Research, U.S.A.; code, 600-401-886). The antibody was diluted 1∶2000 in the TBS and 0.1% Tween.

### Protein Content

Protein contents of all the samples were determined by the Bradford's method using bovine serum albumin (BSA) as standard [100].

### Gene silencing

siRNA sequences targeting Orai1 and Stim1 were purchased by Sigma (esiRNA human Stim1 EHU026401 and esiRNA human Orai EHU120081, respectively) while sequences for the shRNA targeting human TRPC1 were similar to those previously described [101]. Scrambled siRNA and shRNA were used as negative control. Briefly, once the monolayer cells had reached 50% confluence, the medium was removed and the cells were added with Opti-MEM I reduced serum medium without antibiotics (Life Technologies, USA). siRNAs (75 nM final concentration) and shRNA (1 µg/ml final concentration) were diluted in Opti-MEM I reduced serum medium and mixed with Lipofectamine™ RNAiMAX transfection reagent (Invitrogen) pre-diluted in Opti-MEM), according to the manufacturer's instructions. After 20 min incubation at room temperature, the mixes were added to the cells and incubated at 37°C for 5 h. Transfection mixes were then completely removed and fresh culture media was added. The effectiveness in silencing was determined by immunoblotting (see Fig. S10). Knocked out cells were used 72 and 48 hours after transfection for siOrai1 and siStim1, and shTRPC1, respectively.

### Proliferation assays

1×10^5^ EPC-derived cells (1st passage) were plated in 30 mm collagen treated dishes in EGM-2 MV medium with or without 2 to 20 µM BTP-2. We chose this range after preliminary experiments which showed no unspecific or toxic effect for lower or higher concentrations of BTP-2, respectively. Cultures were incubated at 37°C (in 5% CO_2_ and humidified atmosphere) and cell growth assessed every day until confluence was reached in the control cultures (0 µM BAPTA, 0 µM BTP-2 or 0 µM CAI). At this point, cells were recovered by trypsinization from all dishes and the cell number assessed by counting in a haemocytometer. The percentage of growth inhibition by the drugs was calculated by dividing the total number of cells obtained in presence of either BAPTA or BTP-2 or CAI by the number of cells in control experiments and multiplying the ratio by 100.

### *In vitro* tube formation assay

Cells from early passage (P1-P2) EPCs were obtained by trypsinization and resuspended in EGM-2 MV medium. Two ×10^4^ EPC-derived cells per well were plated in Cultrex ® basement membrane extract (Trevigen ®, Gaithersburg, MD, USA)-coated 96 well plates in the presence of BTP-2 (20 µM), BAPTA (30 µM) and CAI (10 µM) (all from Sigma-Aldrich, USA), at 4°C. Plates were then incubated at 37°C, 5% CO_2_ and capillary network formation was assessed starting from 4 to 24 hours later. At least 3 different sets of cultures were performed per every experimental point. Quantification of tubular structures was performed by measuring the total length of structures per field with the aid of the ImageJ software (National Institutes of Health, USA, http://rsbweb.nih.gov/ij/).

### Statistics

As to mRNA analysis, all data are expressed as mean ± SE. The significance of the differences of the means was evaluated with Student's *t* test. In the proliferation assays, results are expressed as percentage (± SD) of growth compared to controls (given as 100% growth), obtained from 3 different sets of experiments, each performed in duplicate. Differences were assessed by the Student t-test for unpaired values. All statistical tests were carried out with GraphPad Prism 4.

### Chemicals

EBM and EGM-2 were purchased from Clonetics (Cell System, St. Katharinen, Germany). Fura-2/AM was obtained from Molecular Probes (Molecular Probes Europe BV, Leiden, The Netherlands). N-(4-[3,5-bis(trifluoromethyl)-1H-pyrazol-1-yl]phenyl)-4-methyl-1,2,3-thiadiazole-5-carboxamide (BTP-2) was purchased from Calbiochem (La Jolla, CA, USA). CAI was a gift from the Drug Synthesis and Chemistry Branch, National Cancer Institute (Bethesda, MD). All other chemicals were obtained from Sigma Chemical Co. (St. Louis, MO, USA).

## Supporting Information

Figure S1 **Average of the Ca^2+^ signals induced by CPA and ATP in endothelial progenitor cells.** Endothelial colony forming cells isolated from the peripheral blood of either healthy donors (black tracings) or patients suffering from renal cellular carcinoma (RCC) were exposed to the so-called “Ca^2+^ add-back” protocol. Briefly, the cells were treated with CPA (10 µM) (A–C) or ATP (100 µM) (D–F) to induce depletion of Ca^2+^ stores in Ca^2+^-free medium (0Ca^2+^) and, subsequently, replaced with Ca^2+^-containing solution so that store-operated Ca^2+^ entry (SOCE) could be measured. In each panel, the Ca^2+^ traces represent the response of cells isolated from three different healthy donors, challenged both with CPA (A–C) and ATP (D–F), and three different patients, again tested both with CPA (A–C) and ATP (D–F). Each tracing is the average of 35–55 cells within one microscopic field. For each panel the two tracings were recorded on the same day.
(TIF)

Figure S2 **Evidence that CPA and ATP activate the same store-operated Ca^2+^ entry pathway in endothelial progenitor cells.** ATP (100 µM) did not trigger any detectable elevation in [Ca^2+^]_i_ upon full activation of SOCE with CPA (10 µM) in both N-EPCs (A) and RCC-EPCs (B). No Ca^2+^ entry occurred in either N-EPCs (C) or RCC-EPCs (D) undergoing the “Ca^2+^ add-back” protocol in the absence of intracellular Ca^2+^ release.
(TIF)

Figure S3 **Down-regulation of inositol-1,4,5-trisphosphate receptors in endothelial progenitor cells isolated from patients affected by renal cellular carcinoma.** Upper panels, mRNA levels were measured by RT-PCR relative to the β-actin internal standard (see Materials and Methods) and the values obtained were reported as ΔCt. Bars represent the mean±SE of at least 4 different experiments each from different RNA extracts. *P<0.05 versus InsP_3_R1 (1-way ANOVA followed by Newman–Keuls's Q test). Lower panels, gel electrophoresis of the PCR products. The PCR products were of the expected size: InsP_3_R1, 180 bp; InsP_3_R2, 158 bp; InsP_3_R3, 173 bp. The specific primers described in Table S2 have been utilized to examine the expression levels of InsP_3_R transcripts. MW: molecular weight marker. Blank: reaction without template.
(TIF)

Figure S4 **Immunohistochemical localization of the Stim1 protein in endothelial progenitor cells.** Stim1 protein was observed in both N-EPCs (A) and RCC-EPCs (B) with a labelling localized within the cytoplasm. Controls in which the primary antibody was substituted by non-immune serum show an absence of labelling (C).
(TIF)

Figure S5 **BTP-2 does not affect SOCE at low concentrations.** Average±SE of the amplitude of SOCE induced by CPA (10 µM) in both N-EPCs (A) and RCC-EPCs (B) in the presence of different concentrations of BTP-2. These doses were ineffective at inhibiting SOCE (p<0.05). In Panel A, n is equal to 63 (black bar) and 36 (white bar) and p = 0.817 for 200 nM BTP-2, n is equal to 63 (black bar) and 38 (black bar) and p = 0.978 for 500 nM BTP-2, and n is equal to 132 (black bar) and 111 (white bar) and p = 0.103 for 2 µM BTP-2. In Panel B, n is equal to 69 (black bar) and 30 (white bar) and p = 0.597 for 200 nM BTP-2, n is equal to 69 (black bar) and 43 (white bar) and p = 0.811 for 500 nM BTP-2, and n is equal to 175 (black bar) and 109 (white bar) and p = 0.597 for 2 μM BTP-2.
(TIF)

Figure S6 **CAI does not increase intracellular Ca^2+^ levels in endothelial progenitor cells.** Acute perfusion of CAI at either 2 µM (A) or 10 µM (B) did not elicit any apparent increase in [Ca^2+^]_i_ in N-EPCs. Each trace is representative of 96 and 138 cells, respectively, isolated from three different donors. Similarly, acute addition of CAI at either 2 µM (C) or 10 µM (D) did not elicit any apparent increase in [Ca^2+^]_i_ in RCC-EPCs. Each trace is representative of 97 and 118 cells, respectively, isolated from three different patients.
(TIF)

Figure S7 **Store-operated Ca^2+^ entry controls tubulogenesis in endothelial progenitor cells harvested from healthy donors.** BAPTA (30 μM), BTP-2 (20 μM), and CAI (20 μM) blocked the assembly into tubulary-like structures of N-EPCs.
(TIF)

Figure S8 **Low concentrations of BAPTA, BTP-2, and CAI do not affect proliferation and tubulogenesis in endothelial progenitor cells.** BAPTA (5 μM), BTP-2 (2 μM), and CAI (2 μM) did not prevent cell growth in both N-EPCs (A) and RCC-EPCs (B). Results are expressed as percentage of growth compared with control (given as 100% growth). C–D, 5 μM BAPTA did not impair tube formation in either N-EPCs (C) or RCC-EPCs (D). E–F, 2 μM BTP-2 had no effect on the assembly of either N-EPCs (E) or RCC-EPCs (F) in a bidimensional tubular network. G and H, 2 μM CAI did not interfere with in vitro tubulogenesis of either N-EPCs (G) or RCC-EPCs (H).
(TIF)

Figure S9 **Genistein prevents SOCE in endothelial progenitor cells.** Genistein (50 µM) fully inhibited SOCE elicited by CPA (10 µM) in both N-EPCs (A) and RCC-EPCs (B). Genistein was applied to EPCs pre-exposed to CPA under 0Ca^2+^ conditions 100 sec before restoration of extracellular Ca^2+^. Each trace is representative of 123 and 142 cells, respectively, isolated from three different donors.
(TIF)

Figure S10 **Decreased expression of Sim1, Orai1, and TRPC1 in silenced endothelial progenitor cells harvested from patients affected by renal cellular carcinoma.** Western blot and densitometry demonstrating a significant reduction in reduced Stim1 (A), Orai1 (B), and TRPC1 (C) protein expression in silenced RCC-EPCs as compared to control cells (C; control vector for A and B and scrambled shRNA for C). The asterisk indicates s p<0.01 (Student's *t*-test). Blots representative of three were shown. Lanes were loaded with 10–30 µg of proteins, probed with affinity purified antibodies and processed as described in Materials and Methods. The same blots were stripped and re-probed with anti-β-actin antibody. Bands of the expected molecular weights were shown.
(TIF)
