# Supplementary figures and images for: Store-Operated Ca2+ Entry Is Remodelled and Controls In Vitro Angiogenesis in Endothelial Progenitor Cells Isolated from Tumoral Patients
Source: PLoS One. 2012 Sep 25;7(9):e42541. doi: 10.1371/journal.pone.0042541 (PMC3458053; doi:10.1371/journal.pone.0042541)

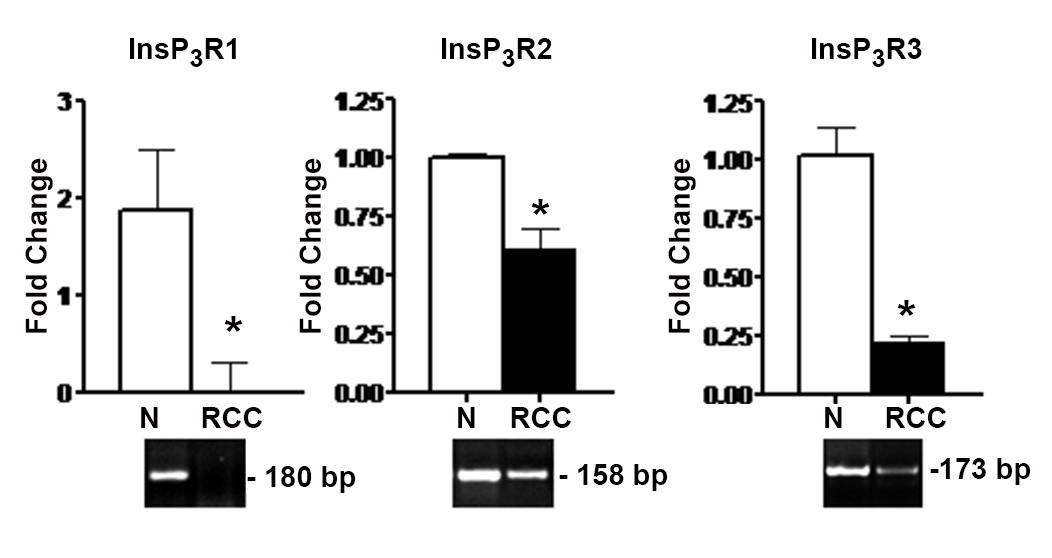

Supplement: Figure S1 — Average of the Ca2+ signals induced by CPA and ATP in endothelial progenitor cells. Endothelial colony forming cells isolated from the peripheral blood of either healthy donors (black tracings) or patients suffering from renal cellular carcinoma (RCC) were exposed to the so-called “Ca2+ add-back” protocol. Briefly, the cells were treated with CPA (10 µM) (A–C) or ATP (100 µM) (D–F) to induce depletion of Ca2+ stores in Ca2+-free medium (0Ca2+) and, subsequently, replaced with Ca2+-containing solution so that store-operated Ca2+ entry (SOCE) could be measured. In each panel, the Ca2+ traces represent the response of cells isolated from three different healthy donors, challenged both with CPA (A–C) and ATP (D–F), and three different patients, again tested both with CPA (A–C) and ATP (D–F). Each tracing is the average of 35–55 cells within one microscopic field. For each panel the two tracings were recorded on the same day. (TIF) [file pone.0042541.s001.tif]

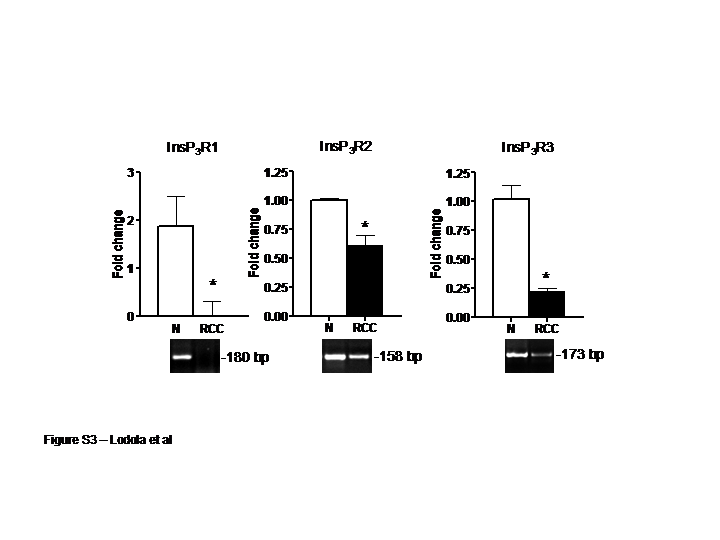

Supplement: Figure S3 — Down-regulation of inositol-1,4,5-trisphosphate receptors in endothelial progenitor cells isolated from patients affected by renal cellular carcinoma. Upper panels, mRNA levels were measured by RT-PCR relative to the β-actin internal standard (see Materials and Methods) and the values obtained were reported as ΔCt. Bars represent the mean±SE of at least 4 different experiments each from different RNA extracts. *P<0.05 versus InsP3R1 (1-way ANOVA followed by Newman–Keuls's Q test). Lower panels, gel electrophoresis of the PCR products. The PCR products were of the expected size: InsP3R1, 180 bp; InsP3R2, 158 bp; InsP3R3, 173 bp. The specific primers described in Table S2 have been utilized to examine the expression levels of InsP3R transcripts. MW: molecular weight marker. Blank: reaction without template. (TIF) [file pone.0042541.s003.tif]

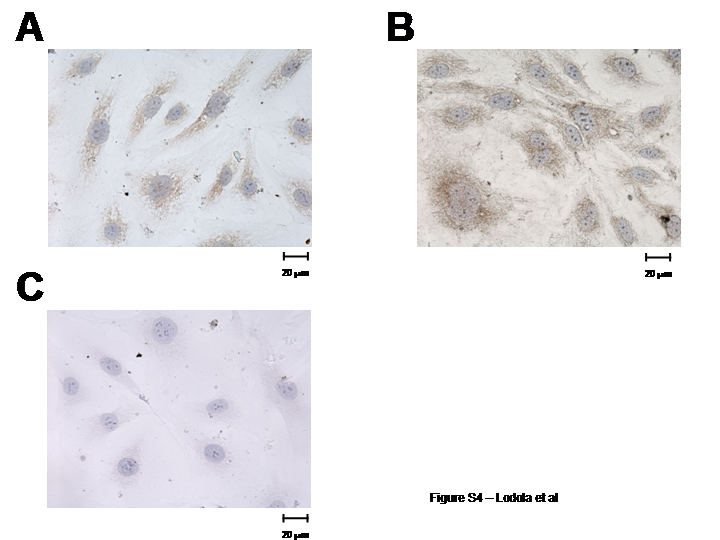

Supplement: Figure S4 — Immunohistochemical localization of the Stim1 protein in endothelial progenitor cells. Stim1 protein was observed in both N-EPCs (A) and RCC-EPCs (B) with a labelling localized within the cytoplasm. Controls in which the primary antibody was substituted by non-immune serum show an absence of labelling (C). (TIF) [file pone.0042541.s004.tif]

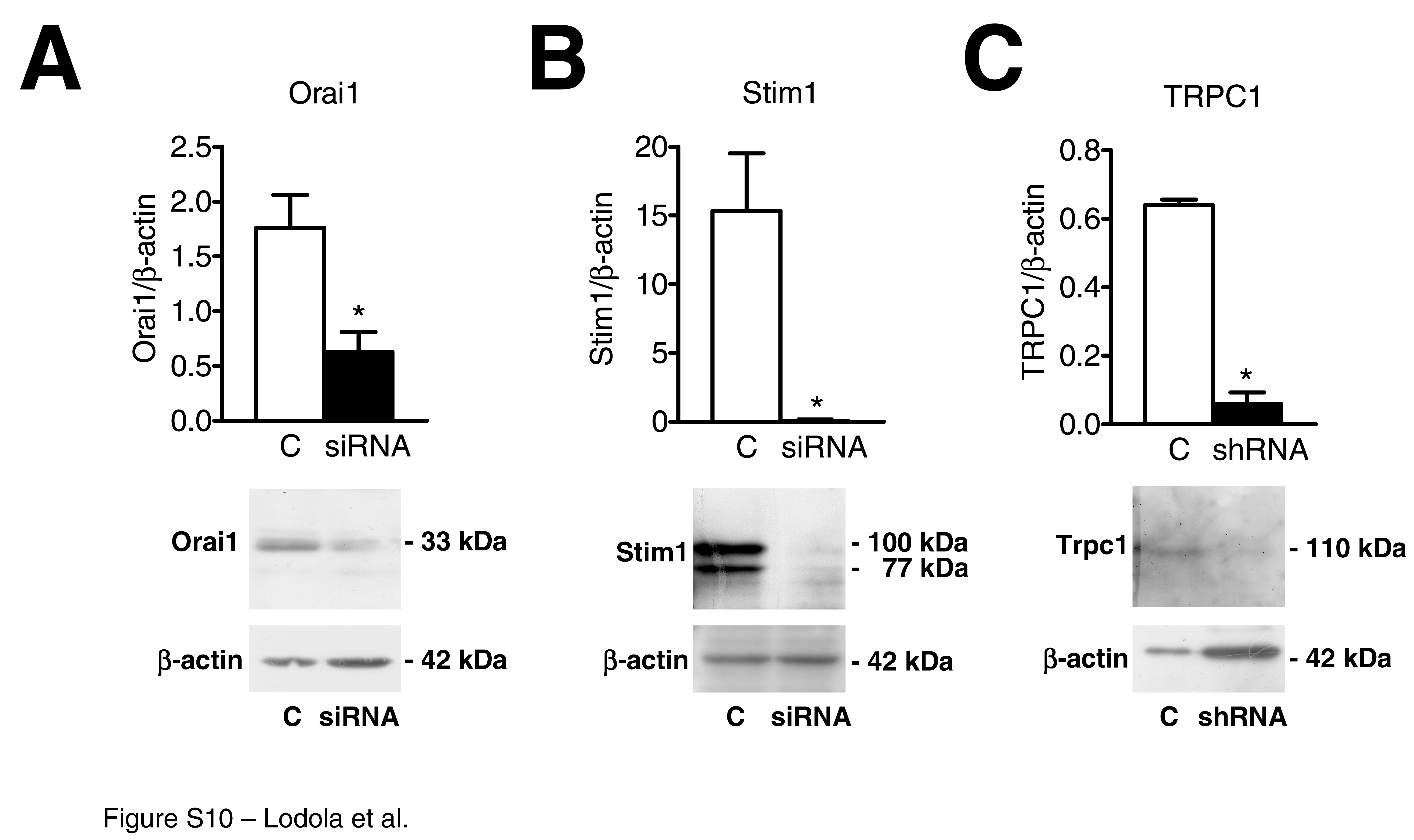

Supplement: Figure S10 — Decreased expression of Sim1, Orai1, and TRPC1 in silenced endothelial progenitor cells harvested from patients affected by renal cellular carcinoma. Western blot and densitometry demonstrating a significant reduction in reduced Stim1 (A), Orai1 (B), and TRPC1 (C) protein expression in silenced RCC-EPCs as compared to control cells (C; control vector for A and B and scrambled shRNA for C). The asterisk indicates s p<0.01 (Student's t-test). Blots representative of three were shown. Lanes were loaded with 10–30 µg of proteins, probed with affinity purified antibodies and processed as described in Materials and Methods. The same blots were stripped and re-probed with anti-β-actin antibody. Bands of the expected molecular weights were shown. (TIF) [file pone.0042541.s010.tif]
